# Molecular Insights into Caffeine Stacking, Partitioning, and Organization in DPPC Lipid Bilayers from Microseconds-Long Molecular Dynamics Simulations

**DOI:** 10.3390/ijms27177704

**Published:** 2026-08-28

**Authors:** Subhalaxmi Das, Nikos Ch. Karayiannis, Supriya Roy

**Affiliations:** 1 School of Applied Sciences, KIIT Deemed to be University, Bhubaneswar, Khurda 751024, Odisha, India; das.subhalaxmi12@gmail.com; 2 Institute for Optoelectronic Systems and Microtechnology (ISOM) and Escuela Técnica Superior de Ingenieros Industriales (ETSII), Universidad Politécnica de Madrid (UPM), José Gutiérrez Abascal 2, E-28006 Madrid, Spain; n.karayiannis@upm.es

**Keywords:** caffeine, drug–membrane interactions, molecular dynamics, DPPC, permeation, atomistic simulation, molecular modelling, lipid membrane, bilayer, potential of mean force, free energy calculation

## Abstract

Caffeine (1,3,7-trimethylxanthine) is a widely consumed psychoactive drug and neurostimulant, yet its molecular organization and permeation behavior in lipid membranes are not fully understood. We employ microseconds-long, united-atom molecular dynamics simulations to investigate caffeine interactions with a solvated DPPC (1,2-dipalmitoyl-*sn*-glycero-3-phosphocholine) bilayer at its fluid phase. Caffeine molecules initially aggregate in the aqueous phase to form ordered stackings, which successively permeate into the membrane. The stacked assemblies gradually dissolve, reaching a stable dispersed state where caffeine molecules preferentially reside near the headgroup–acyl chain interface and orient parallel to the lipid acyl chains, consistent with previous experimental and simulation studies. Simulations initiated with caffeine in the membrane hydrophobic phase converge to the same equilibrium state, indicating a preferred localization at the interface region. Present simulation findings are further supported by free-energy calculations that demonstrate caffeine’s high affinity at the headgroup–acyl chain interface. Caffeine partitioning transiently and slightly reduces bilayer thickness and increases the membrane surface area while enhancing acyl chain stiffness near the hydrophilic part of the membrane. These observed trends are reproducible over different system sizes and independent simulations. Overall, this study provides atomic-level insights into the caffeine permeation process, including its effect on the lipid bilayer structure.

## 1. Introduction

A typical cell membrane is mainly composed of several types of lipids and proteins, and its semi-permeable nature not only controls the selective transport of molecules across it but also protects the cell [1]. Drug–lipid interactions are pharmacologically relevant due to various factors, including drug partitioning into membranes occurring more frequently than non-specific protein binding, accumulation of nonpolar xenobiotics in lipid bilayers and passive transport contributing to drug disposition [2]. Several biophysical methods have paved the way to gaining better insights on drug–membrane interactions, focusing primarily on pharmacokinetics, pharmacodynamics and drug toxicity. Quantitative molecular and thermodynamic descriptions of drug–lipid interactions are frequently obtained using molecular dynamics (MD) simulations, which can access time and length resolutions that are not simultaneously accessible by conventional experimental techniques [3].

Caffeine (1,3,7-trimethylxanthine) exhibits hydrophobic behavior that enables it to permeate through cellular membranes and blood–brain barriers with ease [4]. This marked efficiency makes caffeine an essential central nervous system (CNS) stimulant that increases alertness and reduces sleepiness [5,6]. Consumption of caffeine mitigates liver-related diseases like hepatic steatosis and fibrosis, ultimately lowering the risk of clinical progression to cirrhosis and hepatocellular carcinoma (HCC) [7]. Caffeine, being soluble in both water and lipids, can easily be absorbed from the stomach and small intestine [7]. Caffeine acts as a non-selective antagonist for adenosine G-protein-coupled receptor subtypes, particularly A_1_ and A_2A_, thereby modulating neurobehavioral pathways linked to depression, drug reinforcement, and locomotor sensitization, and offers critical therapeutic insights for Alzheimer’s, Parkinson’s and Huntington’s diseases; multiple sclerosis; heart failure; and cancer [8,9,10,11,12]. Caffeine is also a potential inhibitor for the 3-chymotrypsin-like protease of SARS-CoV-2, which caused the global pandemic in 2019–2023 [5,13]. Apart from being the most widely used psychoactive drug, caffeine exhibits anti-aging curableness, rendering it an essential part of modern medicine [14]. If used as an adjuvant with painkillers, caffeine enhances the analgesic drug properties. It further reduces risks of chronic kidney disease (CKD) and nephrolithiasis [15,16,17] and shows hepatoprotective effects [18].

In a past study, using spectroscopy, molecular docking and simulations, it was found that caffeine could inhibit the digestion of glycoprotein bovine lactoferrin (BLF) and improve its function [19]. Very recently, Alao et al. demonstrated the potential lifespan and health benefits of caffeine from studies on fission yeast [20]. Apart from the health benefits, caffeine plays two primary ecological roles by acting as an allelopathic agent that inhibits the growth of competing vegetation and as a chemical defense mechanism protecting plant tissues from pathogens and animals [21].

At the same time, high doses of intake cause caffeine intoxication, resulting in adverse health effects [22,23]. Caffeine overdoses amplify catecholamine release and cyclic adenosine monophosphate activity, producing widespread metabolic and cardiovascular instability [24,25]. Symptoms like agitation, insomnia, gastrointestinal distress, tachycardia, seizures, and death, in extreme cases, may result from caffeine intoxication [26,27]. It is thus clear that the wide possibilities of caffeine in the fields of medicine, pharmaceutics and general biology render it a prominent and intriguing subject for extensive research.

The active behavior of an amphiphilic compound such as caffeine with the cellular membrane plays a critical role in shaping its overall pharmacological efficacy [28]. Quasielastic neutron scattering (QENS) studies showed that caffeine improves the dynamics of dioctadecyldimethylammonium bromide (DODAB) lipid membrane by acting as a plasticizing agent during the coagel phase; however, in the fluid phase, it restricts lipid dynamics by acting as a stiffening agent [29].

Numerous experimental studies have examined the permeation and dissolution of drugs, including caffeine, into membranes [30,31,32,33,34,35]. In parallel, invaluable insights on drug disposition in the human body and, consequently, on absorption, distribution, metabolism, and excretion (ADME) processes, and on how pharmaceuticals interact with biomembranes, can be provided by simulation and modeling [36]. Towards this, many computational studies, involving primarily MD, have focused on caffeine dynamics in the membrane. The penetration properties of caffeine and its metabolites in 1-palmitoyl-2-oleoylphosphatidylglycerol (POPG) and 1,2-dioleoyl-*sn*-glycero-3-phosphocholine (DOPC) membrane have been described via potential of mean force (PMF) calculations [36]. Based on this, caffeine was preferentially located deeper in the bilayer than the corresponding metabolites, with higher drug concentrations registered for DOPC compared to POPG. The preferred location of caffeine molecules in the membrane was found to be in the water–headgroup interface, as established in past works [37,38]. The free energy profiles of caffeine and DOPC membrane not only showed a minimum at the water–headgroup interface but also revealed a shallow local minimum at the center of the bilayer [36], suggesting a spontaneous partition of caffeine molecules inside the membrane. Combined X-ray diffraction experiments and MD simulations have systematically investigated caffeine and 1-palmitoyl-2-oleoyl-sn-glycero-3-phosphocholine (POPC) membrane interactions [38]. They concluded that water density in the hydrophilic-to-hydrophobic interface increases, resulting in overall dehydration and thickening of the membrane [38]. Additionally, they observed the spontaneous partition of caffeine from the aqueous domain into the membrane. In parallel, an experimental study employing quartz-crystal microbalance and neutron reflectometry techniques determined that caffeine molecules lay parallel to the acyl chains in the hydrophobic region of a POPC bilayer and cannot spontaneously permeate from the aqueous environment into the bilayer [39]. MD simulations of caffeine molecules in aqueous solution systematically showed a tendency to form aggregates, corresponding to stackings of the hydrophobic planar faces [40,41,42].

Previous caffeine–membrane simulation studies have primarily investigated various model membranes, such as POPC, DODAB, and POPG lipid bilayers [29,36]. In the present study, we focus on the interactions of caffeine with a DPPC lipid membrane employing microseconds-long MD simulations. DPPC was chosen due to its biological relevance, as phosphatidylcholines constitute nearly 40–50% of the phospholipids in mammalian cell membranes, making it a suitable model system for eukaryotic membranes. In addition, as a zwitterionic phospholipid, DPPC remains one of the most extensively studied membrane systems for experiments and simulations [43,44,45,46,47,48,49]. Thus, it provides a well-characterized and reliable, yet chemically simple, biological system for investigating the interaction of caffeine with membranes.

In a previous study, we studied the structural and phase behavior of DPPC membrane under lateral pressure, including extreme conditions of compression and stretching [43]. In the present contribution, we investigate the mechanisms governing caffeine–DPPC membrane interactions, with particular emphasis on caffeine clustering, permeation, and eventual dissolution within the lipid bilayer. The significantly prolonged simulation time, reaching 2000 ns (2 μs), ensures, as will be demonstrated in the continuation, capture of the whole permeation process of caffeine, including cluster formation and stacking, penetration, and eventual dispersion into the DPPC bilayer. Additionally, the orientation and positional preference of caffeine, along with the membrane structural alterations induced by caffeine, are systematically examined. We further demonstrate the reproducibility of the present simulation findings through independent simulations, different schemes for the initial caffeine placement and different system sizes.

## 2. Results and Discussion

### 2.1. Molecular Representation/Initial Configurations

**Figure 1:**
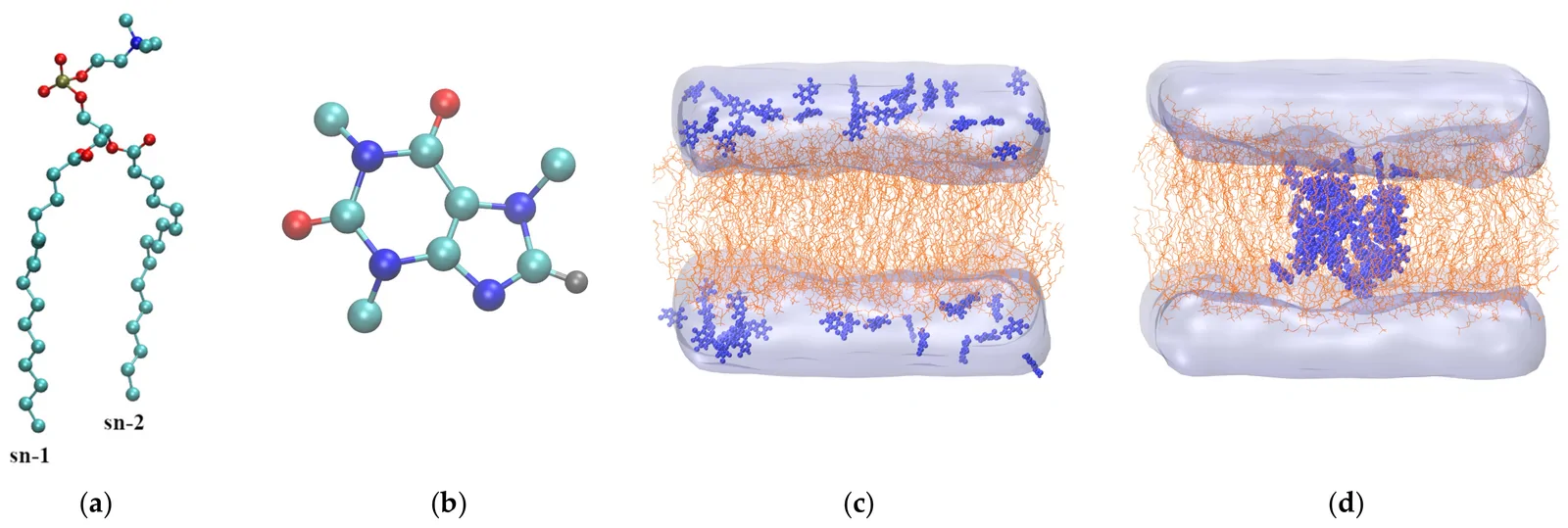
(**a**) A DPPC molecule with two acyl chains (sn-1 and sn-2) is shown in CPK representation, with the carbon, oxygen, nitrogen and phosphorus atoms colored in cyan, red, blue and brown, respectively. (**b**) A caffeine molecule is shown in CPK representation, with carbon, oxygen, hydrogen, and nitrogen colored in cyan, red, grey and blue, respectively. (**c**) Energy-minimized system for the first insertion scheme, where caffeine molecules are dispersed into the water domain. (**d**) System configuration corresponding to the initial part of the trajectory (1 ns) of the second scheme, where caffeine molecules are inserted into the hydrophobic phase. The aqueous solution is represented as a transparent surface. DPPC molecules are represented as orange lines and caffeine atoms are shown as blue spheres in CPK representation for visual clarity. The above color schemes and representations will be followed throughout the manuscript except if otherwise stated. Image created with the VMD software.

Figure 1a,b show a DPPC and caffeine molecule, respectively, in the employed united-atom (UA) representation, while a snapshot of the complete system is shown in Figure 1c. The initial configurations, after the energy minimization, of the complete caffeine + DPPC + water system are shown in Figure 1c,d for the random insertion in the water domain and the droplet-like cluster insertion in the membrane core, respectively.

Videos corresponding to the MD trajectories, showing the evolution of the system for both insertion patterns (in the water domain and in the hydrophobic core of the membrane) and labelled Video S1 and Video S2, respectively, are available as part of the Supplementary Materials.

### 2.2. Caffeine Location

**Figure 2:**
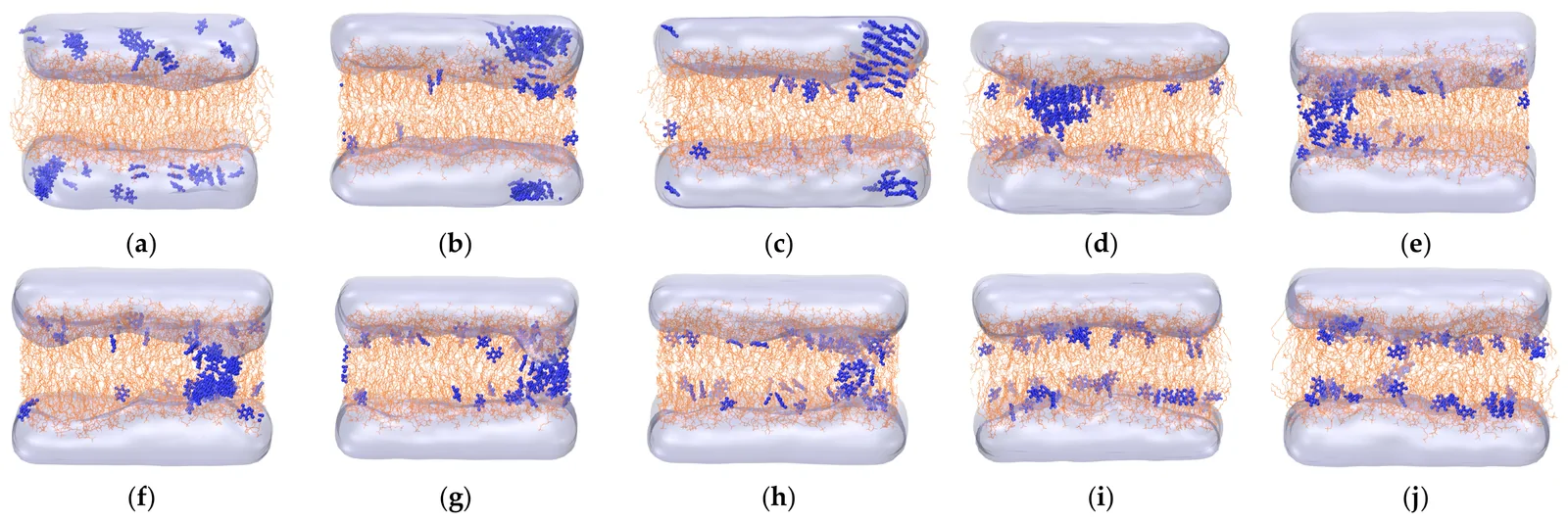
System configurations at various time instances during the NPT MD simulation. (**Top**) From left to right (panels (**a**–**e**)): *t* = 0 (initial configuration as obtained from the 1 ns NVT MD equilibration), 40, 75, 143 and 200 ns. (**Bottom**) From left to right (panels (**f**–**j**)): *t* = 300, 400, 600, 800 and 2000 ns (end of simulation).

The panels of Figure 2 show snapshots (system configurations) at specific time intervals during the production NPT MD simulation covering the whole trajectory of 2000 ns. The time instances have been selected to correspond to different phases of the caffeine permeation into the lipid bilayer. For example, Figure 2a depicts the starting configuration (*t* = 0 ns) after the initial NVT MD equilibration, in which the caffeine molecules remain in the aqueous phase and begin to associate, forming small, spatially dispersed clusters. No caffeine molecule exists in the hydrophobic interior of the membrane. Thus, the simulation is initiated with all caffeine molecules placed in the water layer. As the simulation progresses, caffeine–caffeine and caffeine–water interactions drive aggregation, leading to the formation of a larger caffeine cluster by approximately 40 ns, while a few individual molecules begin to penetrate the membrane, as illustrated in Figure 2b. Subsequently, the aggregated cluster transforms into a columnar stack with most of the caffeine molecules pointing in a single direction, as observed at *t* ≈ 75 ns in Figure 2c.

The stacked caffeine assembly starts to penetrate the water–lipid interface. The gradual permeation continues deeper into the bilayer and adopts a tilted orientation by *t* ≈ 143 ns, with caffeine molecules aligned parallel to the lipid acyl chains (ACs), as shown in Figure 2d. This time marks the transition where no caffeine molecules remain in the aqueous domain of the system. At a later time interval (200 ns ≤ *t* ≤ 400 ns), as seen in Figure 2e–g, transient aggregation occurs near the hydrophobic phase of the membrane, without any visible stacking of the caffeine molecules. This aggregation begins to disperse, as evident in Figure 2h, at *t* ≈ 600 ns, where a significant number of caffeine molecules are detached from the main cluster. The dispersion continues and becomes nearly complete by *t* ≈ 800 ns (Figure 2i), where caffeine molecules are primarily localized in a narrow region along the bilayer normal, very close to the lipid headgroup (HG) in both leaflets of the membrane. Beyond this stage, the spatial distribution remains unchanged until the end of the simulation (*t* = 2000 ns), as reflected in Figure 2j, further marking the end of the clustering–stacking–penetration–dispersion process.

**Figure 3:**
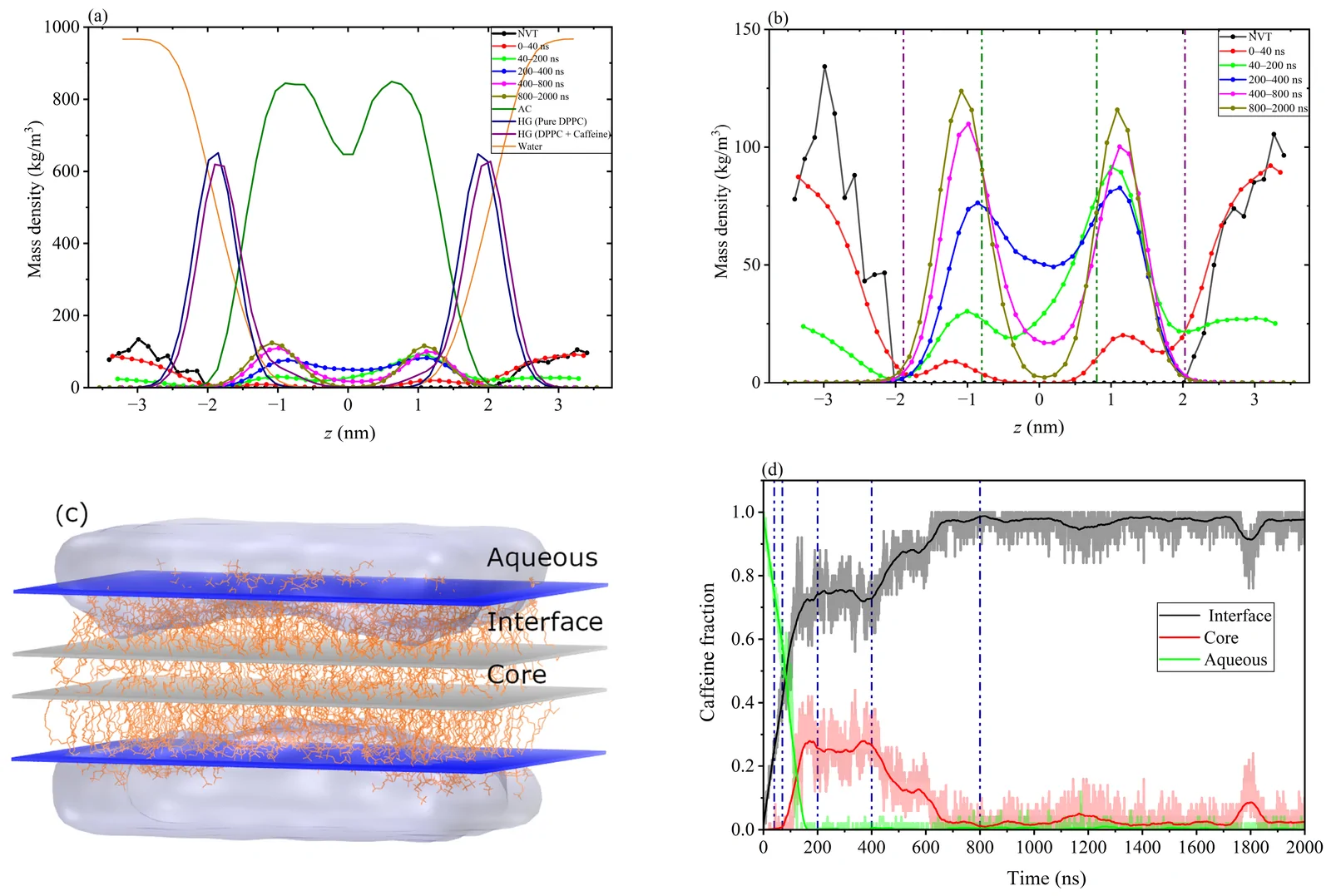
(**a**) Mass density profile for the center of mass of caffeine molecules, and the head groups (HGs) (pure DPPC system in navy blue and caffeine + DPPC in purple) and acyl chains (ACs) for the caffeine + DPPC system (olive) and of water (orange). The HG, AC and water profiles correspond to the last part of the simulation trajectory (800–2000 ns). Different profile curves for caffeine (lines with filled circle symbols) correspond to different time intervals, including the initial equilibration NVT simulation. (**b**) Mass density profile focusing exclusively on the caffeine molecules. The vertical purple and olive dashed-dotted lines mark the peaks of the HG and AC mass density profiles, respectively, as identified in panel (**a**). The peaks correspond to the last part of the simulation: 800–2000 ns for the current caffeine + DPPC system and the last 400 ns for the pure DPPC system from our previous study [43]. (**c**) Schematic of the DPPC membrane + water system being portioned into three regions: aqueous (|*z*| ≥ 2 nm), interfacial mixed (0.5 nm ≤ |*z*| ≤ 2 nm), and hydrophobic membrane core (|*z*| ≤ 0.5 nm). The grey and blue planes define the transitions between the different regions. (**d**) Caffeine fraction as a function of time in aqueous, interfacial and core regions, as identified in panel (**c**). Thick and thin lines correspond to smoothed and instantaneous values, respectively. The blue dot-dashed vertical lines are placed at *t* = 40, 75, 200, 400 and 800 ns to indicate the distinct permeation phases.

The mass density profile of caffeine, utilizing the center of mass of each molecule as a reference, along the bilayer normal (*z*-axis) is calculated over different time intervals (Figure 3a). To determine the spatial distribution of caffeine molecules relative to the membrane and the aqueous solution, in addition to the caffeine data, the density profiles of the HG and ACs for DPPC and of water are also present in panel (a). For visual clarity and to highlight the differences between the different steps of the process, Figure 3b hosts the mass density profiles only for caffeine.

Focusing on the DPPC and water components, the following conclusions can be drawn from the corresponding density profiles: (a) for coordinates *z* ≤ −2 nm and *z* ≥ +2 nm, the domain is occupied solely by water molecules; (b) the spatial intervals −2 nm ≤ *z* ≤ −0.5 nm and +0.5 nm ≤ *z* ≤ +2 nm correspond to the interface formed by water molecules and the hydrophilic head of the DPPC, which comprises a mixture of headgroup (HG) and acyl chain (AC) segments and accounts for ~75% of the membrane; and (c) the interval −1 nm ≤ *z* ≤ +1 nm marks the hydrophobic core of the DPPC assembly where no water molecules reside, composed of AC tail carbons and constituting the remaining ~25% of the membrane. Additionally, by comparing the HG profiles of the system with (present work) and without ([43]) caffeine molecules in the water–membrane interface, the HG profile is more extended and slightly penetrates the interior of the hydrophobic lipid core in the presence of caffeine. This is evidenced by the HG concentration in the intervals −1 nm ≤ *z* ≤ −0.5 nm and +0.5 nm ≤ *z* ≤ +1 nm being significantly higher in the case of the caffeine-loaded system compared to the pure DPPC + water one. Based on the above, Figure 3c shows a schematic of the total system, with horizontal planes identifying the limits of the three different domains (aqueous, interfacial and core).

During the initial phase, caffeine remains in the aqueous region, with no molecules penetrating the interface or the membrane, as demonstrated by the corresponding density curve in Figure 3b.

During the initial time intervals of 0–40 ns and 40–200 ns of the main production simulation, a gradual increase in caffeine density between the headgroup planes is observed, indicating partial penetration of caffeine into the membrane, while the main fraction of molecules remains in the solvent phase. This trend corresponds to the early stage of membrane permeation by caffeine molecules. The interval of 200–400 ns shows that no caffeine exists in the aqueous domain and thus marks a transition where caffeine has successfully penetrated the water–membrane interface. In parallel, many caffeine molecules are located deep in the hydrophobic core of the DPPC molecular assembly, as evidenced by the almost flat mass density profile in the range of −1 nm ≤ *z* ≤ +1 nm. The penetration process of the caffeine molecules from the aqueous solution into the membrane is completed by ~200 ns, as the mass density profiles of the caffeine molecules for the later instances (200–400, 400–800, and 800–2000 ns) show almost zero density in the region of 2 nm≤z≤ −2 nm. Caffeine molecules reside exclusively in the hydrophobic core of the membrane at later time intervals (400–800 ns and 800–2000 ns), and their mass density profile is significantly different compared to the earlier one (200–400 ns): in the latter two, symmetrically placed peaks appear around −1.1 and +1.1 nm, becoming even more pronounced near the end of the simulation. In parallel, the caffeine population in the central volume of the membrane core (−0.5 nm ≤ z ≤ +0.5 nm) progressively diminishes. This is in sharp contrast to the almost flat profile adopted by caffeine molecules once they permeate the membrane. The observed symmetric peaks, formed at approximately ± 1.1 nm, correspond to planes located between the lipid HG and ACs, representing the headgroup–acyl chain interfacial region and indicating a preferred localization of caffeine at this interface. The decay of caffeine density at the bilayer center to a value of nearly zero further suggests that the penetrant molecules clearly avoid the highly hydrophobic core of the membrane and align with the lipid head groups. The highly favored caffeine localization at the HG–AC interface, as clearly seen in the present MD simulations using the L-B force field, are further supported by the PMF calculations, as will be demonstrated next.

The fraction of caffeine molecules in the three identified regions (aqueous, interfacial and core) of the total system is further calculated and monitored throughout the simulation, as seen in Figure 3d. The final configuration of the NVT simulation, which serves as the initial configuration for the production MD, exhibits a caffeine fraction close to unity in the aqueous region, indicating that nearly all caffeine molecules remain initially solvated. This fraction decreases approximately linearly with time, dropping to zero by *t* ≈ 200 ns, consistent with the progressive penetration of caffeine into the membrane. Concurrently, the caffeine fraction in the (HG–AC–water) interface increases in a complementary manner and reaches a plateau over a similar time scale, indicating substantial accumulation in this mixed region. In contrast, the fraction of caffeine in the hydrophobic core remains negligible until ≈50 ns, after which it increases gradually, reaching a transient plateau around ≈200 ns and persisting until ~400 ns, coexisting with the population plateau observed for the interfacial domain. Beyond this point, the caffeine fraction in the hydrophobic core (−0.5 nm ≤ *z* ≤ +0.5 nm) of the membrane decreases steadily, approaching zero by ~800 ns, accompanied by a corresponding increase in the HG–AC mix region, approaching a value close to unity. This trend demonstrates that while caffeine molecules reach the bilayer center during the early penetration process (*t* ≈ 50 ns), they reside there only transiently, before moving to the HG–AC mix region.

Based on the above, the central volume of the bilayer is not thermodynamically favorable for sustained caffeine localization; rather, the transient population reflects an early step where the caffeine molecules self-assemble into a large cluster, as visualized in Figure 2d–f and as will be demonstrated later in the cluster analysis. This caffeine aggregate temporarily reaches the hydrophobic core of DPPC before its eventual dissolution. At longer times, caffeine eventually redistributes toward the interfacial region, consistent with the density profile analysis, indicating preferential localization near the headgroup–acyl chain (HG–AC) interface.

The conclusions described above, as drawn from both the density profile and the temporal evolution of caffeine fractions, consistently indicate that caffeine molecules first self-assemble, then penetrate and eventually disperse preferentially at the interface between the HG and AC regions. This interfacial preference is in excellent qualitative agreement with past studies. Previous MD simulations [37] in DOPC membrane demonstrated that the free energy minimum lies beneath the HG region at a distance of 1.1–1.7 nm from the bilayer center. Khondker et al. [38] reported caffeine localization near the head group–tail group interface of the POPC bilayers, at |*z*|-values of 1.5 and 1.7 nm from simulations and experiments, respectively. The experimental studies by Tavagnacco et al. [39] on supported POPC bilayers found caffeine molecules to orient parallel to the acyl chains, and residing at 0.78 nm from the bilayer center just below the HG region, qualitatively matching our results. However, they also found that the caffeine could not permeate into the membrane from the aqueous solution.

Although DPPC and POPC are both phosphatidylcholine (PC) lipids, direct comparison between these systems should be carried out with caution because of their differences in acyl chain composition, lipid ordering, membrane thickness and caffeine concentrations. Nevertheless, the present results reveal a comparable localization behavior for the caffeine in DPPC membrane, with caffeine accumulating near the interface formed between the HG and AC segments. The preferred dispersion position along the *z*-axis occurs at approximately 1.1 nm from the center, as evidenced by the density profiles (Figure 3b).

### 2.3. Cluster Analysis

It is apparent from the snapshots in Figure 2 that caffeine aggregates in a stacked form, while in the aqueous solution, it penetrates as a single stack into the membrane and eventually disintegrates into smaller clusters or individual molecules, all localized in the HG–AC interface. To provide quantitative information about caffeine aggregation and dispersion over time, we carry out cluster analysis focusing on caffeine molecules. Each caffeine molecule is represented here through its center of mass (C.o.M.).

**Figure 4:**
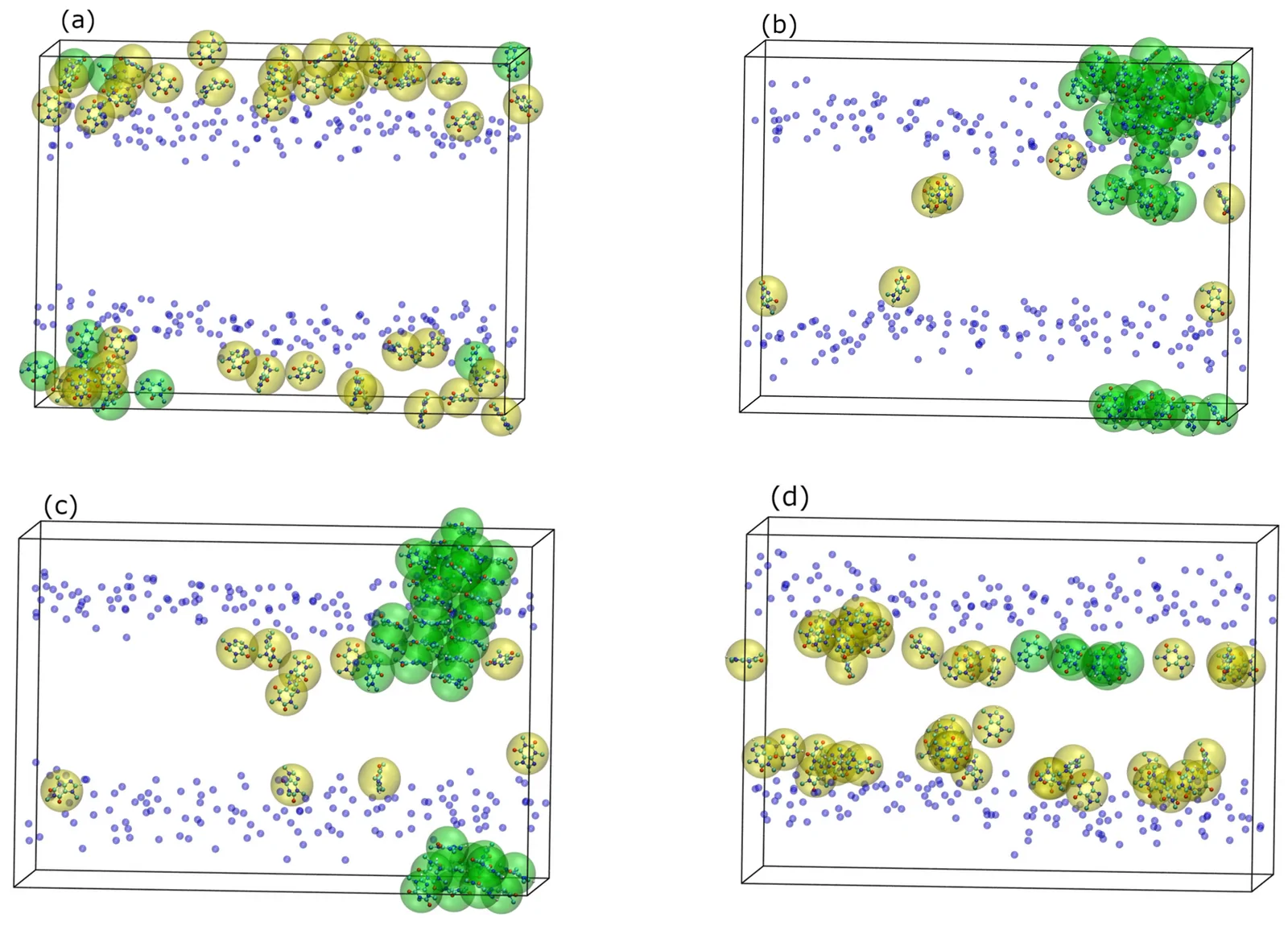
Caffeine clustering, stacking and dispersion during membrane permeation. The water and DPPC are purposefully hidden to increase the clarity of the formed clusters (for a more detailed representation, please see Figure S1 in Supplementary Materials). Caffeine molecules belonging to the largest cluster are shown in green spheres, and rest of the molecules appear in yellow spheres. In both cases, the radius of the spheres coincides with the threshold distance employed for the cluster analysis. The small blue spheres are the nitrogen (N) atoms of the DPPC headgroups. (**a**) Configuration after energy minimization. (**b**) Maximum size of cluster for *t* = 40 ns. (**c**) Ordered cluster (caffeine stacking) at *t* = 75 ns. (**d**) Dispersion of caffeine molecules near the headgroups of both leaflets at the end of the simulation (*t* = 2000 ns).

**Figure 5:**
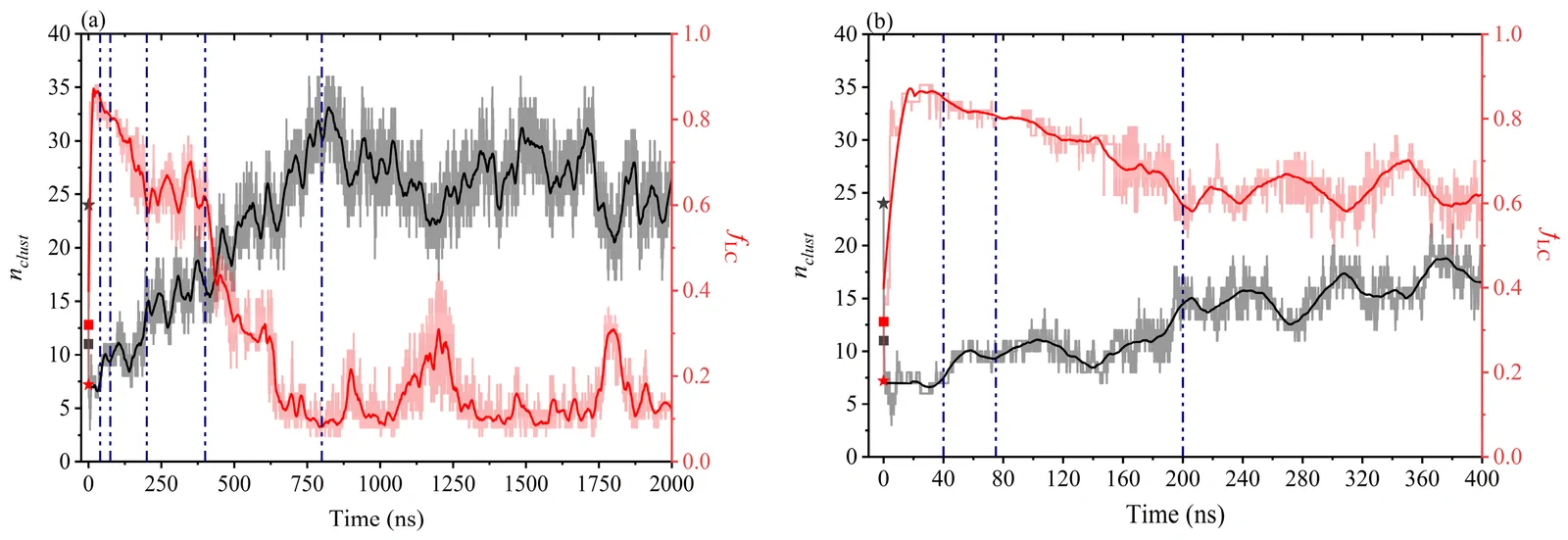

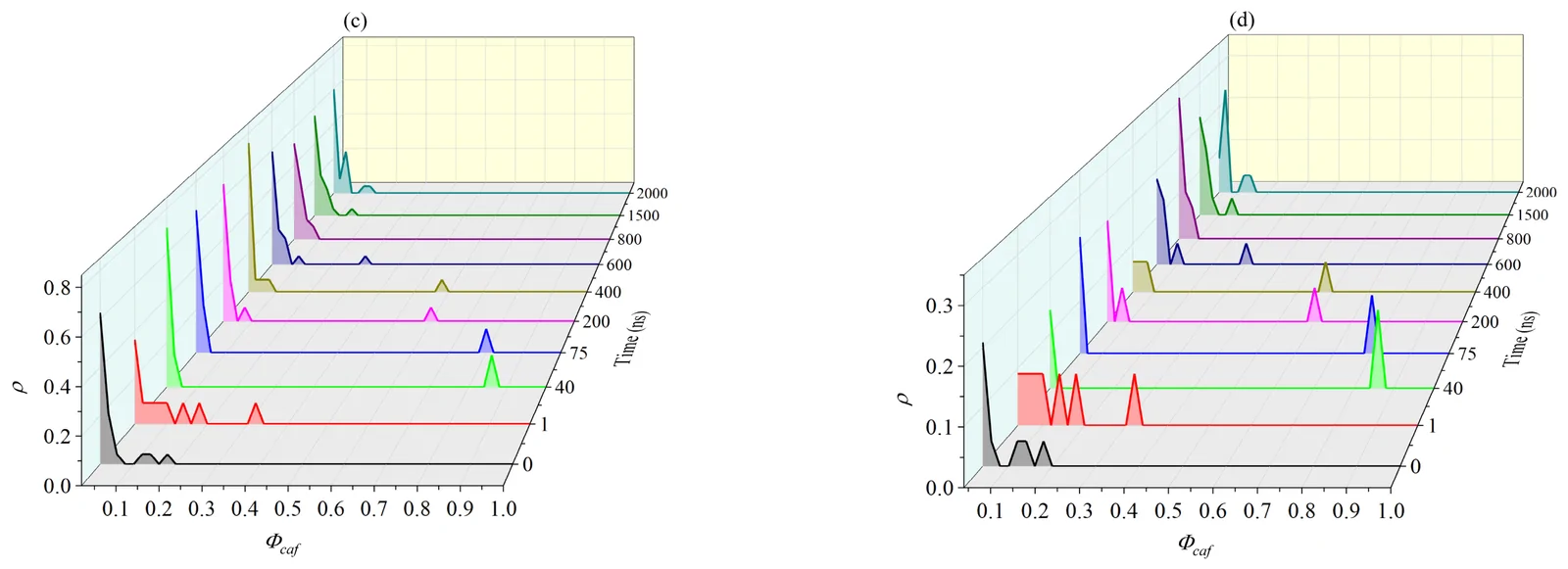
(**a**) Number of distinct clusters (black line, left *y*-axis), *n_clust_*, and caffeine fraction in the largest cluster (red line, right *y*-axis), *f_LC_*, as a function of time. Thin and thick lines correspond to instantaneous (raw) and smoothed values, respectively. Asterisk and square symbols denote the cluster data as extracted from the system configuration after energy minimization and the 1 ns-long equilibration NVT MD simulation, respectively. The blue dot-dashed vertical lines are placed at *t* = 40, 75, 200, 400 and 800 ns to indicate the distinct permeation phases. (**b**) Same as in panel (**a**), focusing on a narrower initial time range. (**c**) Probability distribution of cluster size (measured in fraction of caffeine molecules), *ϕ_caf_*, at different time instances. (**d**) Same as in panel (**c**) but excluding the clusters of size 1 (i.e., individual caffeine molecules).

A cluster is defined as several inter-connected caffeine molecules, where connectivity is established if the distance between any pair of molecules is ≤1 nm. The minimum cluster size is defined as one, i.e., a cluster can be composed of a single isolated caffeine molecule. The algorithm utilized for the cluster analysis is the same as the one presented in [50]. Figure 4 shows the cluster formation of the caffeine molecules as the simulation evolves, where the caffeine molecules in the largest cluster are shown inside green spheres, whereas the rest are shown inside yellow ones. The sphere radius has been selected to coincide with the distance threshold adopted for the cluster analysis. A detailed representation of the same snapshots is provided in Figure S1 (Supplementary Materials). Using this geometrical analysis, we calculate the number of distinct clusters, $n_{clust}$, and the caffeine fraction in the largest cluster, $f_{LC}$, shown in Figure 5a,b. $f_{LC}$ is defined as the number of caffeine molecules in the largest cluster (which is equivalent to the size of the cluster) divided by the total number of caffeine molecules. This parameter is therefore bounded between 0.02 (corresponding to a single caffeine molecule) and 1 (corresponding to a cluster containing all 50 caffeine molecules).

Additionally, we calculate the probability of cluster size, ρ, as a function of caffeine cluster size, $\phi_{caf}$, and plot it in Figure 5c,d. Here, $\phi_{caf}$ is defined as the fraction of caffeine molecules in the clusters. Therefore, $\phi_{caf}$ also ranges from 0.02 to 1.

Figure 5a shows the time evolution of the number of distinct clusters, *n_clust_*, and the caffeine fraction in the largest cluster, *f_LC_*, during the MD simulation. Also shown in the same graph are the corresponding data after the energy minimization (filled asterisk) and the 1 ns-long equilibration NVT simulation (filled square). With respect to the initial system configuration, the number of distinct clusters is elevated (*n_clust_* = 24), while the size of the largest cluster is rather limited (*f*_LC_ = 0.18), as shown in Figure 5a,b. In parallel, the cluster size distribution, as seen in Figure 5c, reveals further that the probability of having clusters with a size larger than 1 is very low. This can be confirmed visually in Figure 4a, which shows a rather uniform dispersion of caffeine molecules in the aqueous environment, leading to very small and isolated clusters. The output of NVT simulation shows a drop in the number of distinct clusters, *n_clust_* = 11, while the caffeine fraction in the largest cluster, *f*_LC_, increases to 0.32, which is almost double the size of the preceding configuration, corresponding to energy minimization. This significant increase in the size of the largest cluster marks the onset of caffeine aggregation. As time progresses, the number of distinct clusters reduces to the smallest value of *n_clust_* = 7 at around *t* = 40 ns, while the size of the largest cluster reaches its maximum value, with *f*_LC_ = 0.86 associated with the maximum value of *ρ* ~ 0.14, indicating that it incorporates almost all caffeine molecules, which is also verified visually in Figure 2b and Figure 4b.

As the simulation evolves, the number of distinct clusters increases gradually, while the size of the largest cluster drops at a similar rate. For example, *ρ* drops from ~0.14 (*t* = 40 ns) to approximately 0.066 at *t* = 200 ns, demonstrating a significant size reduction for the caffeine cluster. In the interval of 200–400 ns, *n_clust_* and *f*_LC_ both exhibit stable behavior within relatively small fluctuations, indicative of a sustained overall cluster formation. This stable behavior can be correlated with the transient populated phase in the central part of the membrane, which occurs at the same time interval. For example, the size probability values that correspond to the largest cluster at *t* = 200 and 400 ns adopt nearly identical values: 0.066 and 0.062, respectively.

The time interval of 400–800 ns shows a significant drop in the size of the largest cluster from 0.6 at 400 ns to 0.08 at 800 ns, marking its gradual collapse. Eventually, in the final phase of the simulation (800–2000 ns), a plateau is reached for both the *n_clust_* and *f*_LC_. This plateau corresponds to a limited cluster size (*f*_LC_ < 0.15), which is further subjected to fluctuations as isolated molecules become linked and detached over time. As a final note, the minimum cluster size does not drop below a specific value, as the volume of the hydrophobic and interfacial core is not sufficiently large to accommodate full dispersion of the caffeine molecules. In significantly larger (along the *x*–*y* dimensions) system realizations, the cluster size could be reduced to even lower values.

### 2.4. Caffeine Orientation

**Figure 6:**
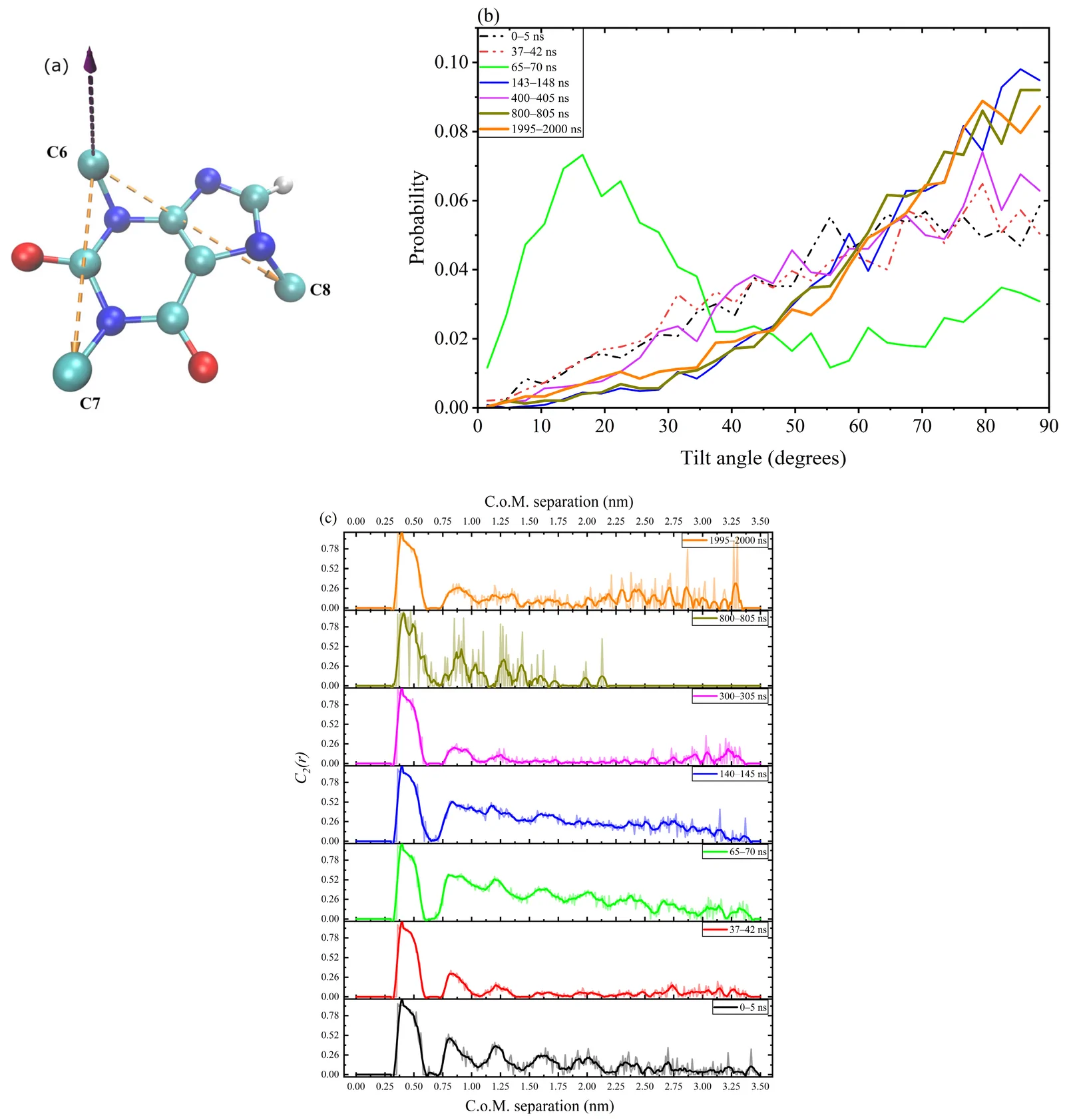
(**a**) Schematic representation of the caffeine molecule where the orange dashed arrows drawn correspond to the vectors connecting carbon pairs C6–C7 and C6–C8. The violet dashed arrow corresponds to the vector normal to the plane defined by the C6–C7 and C6–C8 vectors. (**b**) Probability distribution of caffeine tilt angle at various time intervals. (**c**) Nematic correlation function of caffeine molecules belonging to the largest cluster at various time intervals.

The orientation of caffeine molecules is evaluated by calculating the tilt angle relative to the bilayer normal (*z*-axis). For each caffeine molecule we define two vectors, connecting the carbon pairs C6–C7 and C7–C8, as seen in the sketch of Figure 6a, and the vector normal to their plane is computed. The angle formed by this normal vector and the bilayer normal is defined as the caffeine tilt angle. Thus, the caffeine orientations parallel and perpendicular to the membrane surface correspond to a tilt angle of 0° and 90°, respectively. The tilt angle distribution at various time frames is plotted in Figure 6b. At the beginning (*t* ≤ 5 ns), it corresponds to caffeine dispersion in the aqueous environment and shows an increasing trend for higher tilt angles, reaching a plateau beyond 50°. This indicates a higher preference for caffeine molecules to adopt orientations parallel to the bilayer normal.

During the time frame of 37–42 ns, which corresponds to extensive clustering (see also Figure 2b and the cluster analysis data in Figure 5a), the distribution remains quantitatively similar to the initial phase. The broad distribution observed for the tilt angles indicates that, despite significant aggregation, the caffeine molecules still do not adopt a preferential stacking conformation. However, a marked change is observed in the continuation, as for the 65–70 ns interval, the distribution shows a broad peak in the low angle range of 15 to 35°, which corresponds to columnar stacking in which the molecular normal is nearly parallel to the bilayer normal. Deviations from the observed peak and the corresponding stacking arrangements can be attributed primarily to individual caffeine molecules that have already penetrated the lipid membrane, as clearly seen in Figure 2c. During 143–148 ns, a significant increase in the population of angles near 90° is observed, arising from columnar stacks aligned along the bilayer normal. These conformations are consistent with the snapshot in Figure 2d.

During 400–405 ns, the distribution again shows more uniform distribution, which could be attributed to the transient aggregation near the hydrophobic core, as depicted in Figure 2h. As discussed earlier, the process is completed around 800 ns as caffeine reaches the HG–AC interface. Accordingly, during the final equilibrium phase (800–2000 ns), the tilt angle distribution also becomes stable, demonstrating a peak at around 90° for both the time windows, at 800–805 ns and 1995–2000 ns. The persistence of such a pronounced peak beyond 800 ns further indicates that, in the equilibrated state, caffeine molecules preferentially orient with their molecular plane perpendicular to the bilayer normal, i.e., approximately parallel to the lipid acyl chains.

### 2.5. Nematic Correlation Function

Caffeine molecules are known to form stacked aggregates in aqueous solution, and previous experimental [51,52] and simulation studies [53] have characterized their interplanar separation and preferred stacking geometries. As shown in the snapshots of Figure 2, in the present simulation study the caffeine molecules exhibit pronounced stacked arrangements near the water–bilayer interface, which are particularly evident during the membrane entry phase. As the simulation progresses, the aggregate size gradually decreases, and the long-range stacking correlations diminish. To quantify the extent of orientational correlation between caffeine molecules as a function of intermolecular separation r, we compute the nematic correlation function, defined as $C_{2}\left(r\right)={\langle P_{2}(\hat{u_{i}}.\hat{u_{j}})\rangle }_{\left|r_{ij}\right|=r}$, where, $P_{2}\left(\hat{u_{i}}.\hat{u_{j}}\right)=\frac{1}{2}\left(3{cos}^{2}\theta -1\right)$ is the second-order Legendre polynomial, and $\hat{u_{i}}$ and $\hat{u_{j}}$ are the unit orientation vectors of caffeine molecules *i* and *j*, respectively [54]. The orientation vector of the caffeine molecule is essentially the same vector that is used for measuring the tilt angle. The cosine term corresponds to the angle, *θ*, between the two molecular orientation vectors. The nematic correlation function, following a similar approach for the orientational correlation function, as presented in [55], is evaluated by averaging over all molecular pairs whose center-of-mass (C.o.M.) separation lies within the interval r+δr, followed by ensemble averaging over all configurations for a given time window. Here, δr is set as 0.1 nm. Thus, $C_{2}(r)$ measures how strongly two caffeine molecules are orientationally aligned to each other as a function of their separation distance, reflecting the degree of stacking present in the system. $C_{2}\left(r\right)$ is calculated only for the caffeine molecules composing the largest cluster of each configuration.

The nematic correlation function $C_{2}$ is calculated over time intervals of 5 ns (practically 50 successive frames) at different segments of the trajectory, and the results are reported in Figure 6c. Owing to the limited number of configurations per window and the limited number of caffeine molecules in the largest cluster, the resulting $C_{2}$ profiles exhibit noticeable statistical noise. Therefore, a smoothing procedure is applied to reduce fluctuations and improve clarity in the data representation.

At the initial stage of the simulation (0–5 ns), $C_{2}$ displays four to five successive peaks, indicative of multiple interplanar stacked aggregates in the aqueous solution and marking the onset of aggregation. The pronounced yet gradually decaying peaks suggest strong interplanar packing within small aggregates, as shown in Figure 2a. By 37–42 ns, both the peak intensity and periodicity are significantly reduced, with oscillations nearly vanishing beyond the third peak, suggesting disruption of extended stacked arrangements within the largest cluster. This trend is consistent with the broader tilt-angle distribution observed over the same interval, reflecting diminished orientational order.

In contrast, during 65–70 ns, $C_{2}$ exhibits persistent oscillations extending over six to seven peaks with smaller decay, indicating long range correlations and signifying well-defined larger multilayer stacking. This behavior agrees with the stacking conformation of the largest caffeine cluster (Figure 2c), which shows pronounced interplanar organization. At 140–145 ns, sustained correlations remain, albeit slightly attenuated compared to 65–70 ns, consistent with the conformation of the largest caffeine aggregate being embedded within the membrane and adopting multilayer stackings primarily in a parallel-to-AC orientation, as supported by the prominent peak near 90° in the tilt angle distribution (Figure 6b). In the later intervals (300–305, 800–805, and 1995–2000 ns), $C_{2}$ shows markedly reduced ordering, characterized by weaker oscillations and fluctuations around low values, signaling the absence of extended stacking caffeine conformations.

The interplanar separation between two stacked caffeine molecules is estimated from the position of the first peak in the $C_{2}\left(r\right)$ correlation function. For all time intervals examined, this peak is located at around 0.39–0.41 nm. The interplanar distances obtained in the present study are in good agreement with past reports. In a previous experimental study, the interplanar distances of the caffeine dimers calculated for anhydrous caffeine crystals were found to vary from 0.322 nm to 0.347 nm [53]. Furthermore, Density Functional Theory (DFT) simulations of caffeine in aqueous solution reported an average interplanar distance of 0.39 nm [51]. Another MD simulation study, of caffeine in aqueous NaCl salt solution, reported the formation of higher-order caffeine clusters with a spacing of 0.37 nm [56]. The values obtained here, in the range of 0.37–0.45 nm, fall within the experimentally and computationally reported range.

### 2.6. Effect of Caffeine on the Membrane Bilayer

Small amphiphilic molecules are known to modulate the physicochemical properties of lipid bilayers, which can influence membrane protein function [57]. These molecules perturb key membrane characteristics, such as elasticity, curvature, and thickness, thereby altering the energetic landscape associated with protein conformational changes and potentially modulating their activity. Consequently, understanding drug–membrane interactions is essential for evaluating both toxicity and therapeutic efficacy [58]. In this context, the present study examines the effect of caffeine on membrane structure by quantifying changes in membrane surface area, lipid chain ordering, and bilayer thickness.

#### 2.6.1. Surface Area and Bilayer Thickness

**Figure 7:**
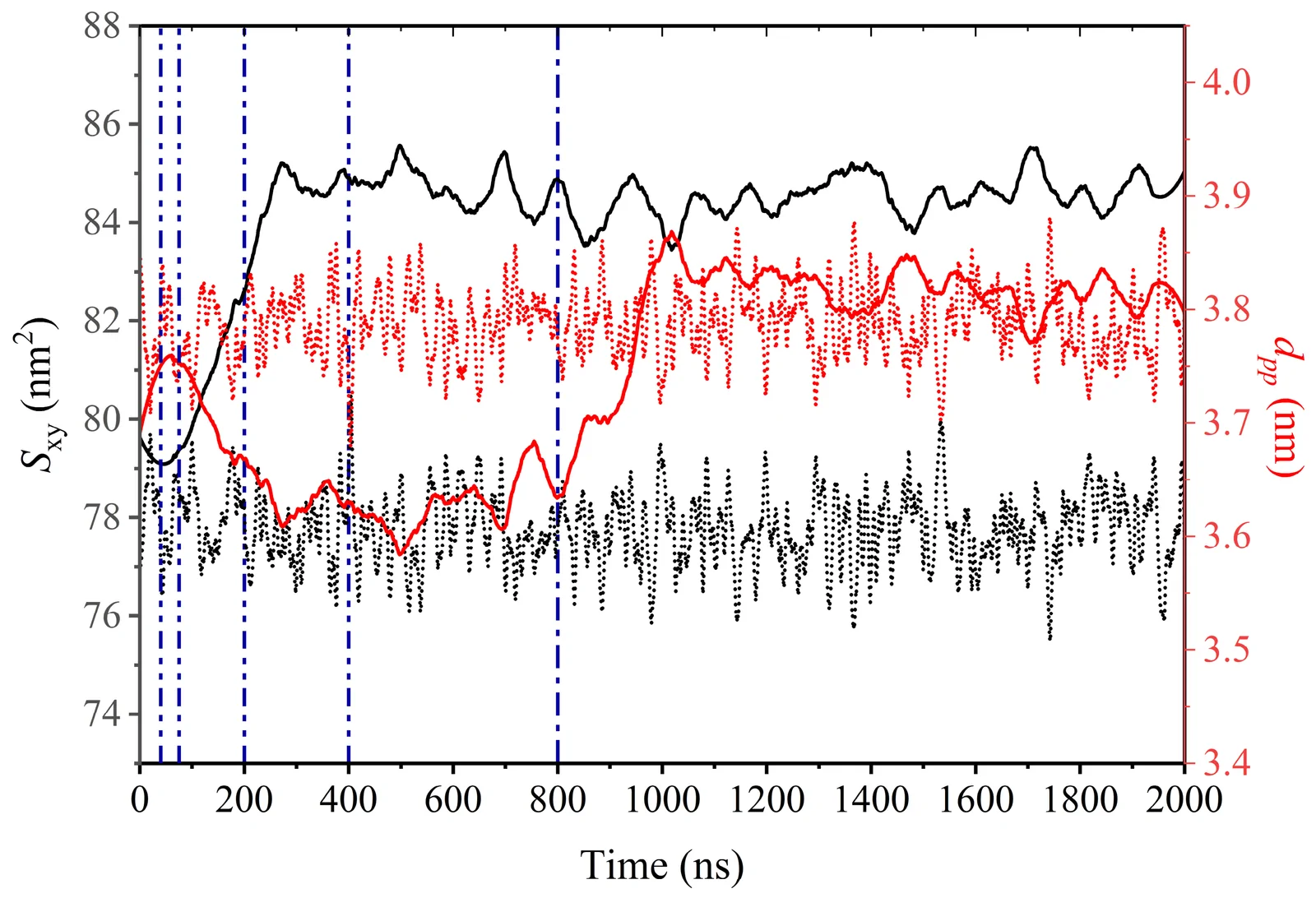
Variation in surface area, *S_xy_* (left *y*-axis), and bilayer thickness, *d_pp_* (right *y*-axis), as a function of time. Solid and dotted lines correspond to the caffeine-loaded and pure DPPC membrane, respectively. The blue dot-dashed vertical lines are placed at *t* = 40, 75, 200, 400 and 800 ns, marking the different phases of the caffeine permeation process.

The surface area of the membrane is calculated as $S_{xy}=L_{x}L_{y}$, and its time evolution is shown in Figure 7. $L_{x}$ and $L_{y}$ are the simulation box lengths in the *x*–*y* membrane surface. They have also been reported as the corresponding data for the caffeine-free (pure) DPPC membrane in water [43]. The surface area increases nearly linearly with time, from an initial value of *S*_xy_ ≈ 78 nm^2^ to approximately 84 nm^2^ by *t* ≈ 300 ns, corresponding to an increase of about 8%. After this transient increase, the membrane surface area stabilizes for the remainder of the simulation.

Cluster analysis indicates that maximum caffeine aggregation occurs by *t* ≈ 40 ns, which evolves into a stacked configuration by *t* ≈ 65 ns, as evident in the snapshots in Figure 2b,c. Due to the stacked organization, fewer caffeine molecules are exposed to the membrane surface, resulting in an almost constant $S_{xy}$ during the early stages of the simulation (*t* ≤ 90 ns), indicating that the surface area is only weakly affected. As the stacked aggregates begin to disperse, a larger number of caffeine molecules become exposed to the membrane surface, contributing to the overall expansion of the membrane surface.

The bilayer thickness (*d_pp_*), also presented as a function of time in Figure 7, is calculated as the distance between the average *z*-coordinate of the phosphorus (P) atoms of the headgroups in the top and bottom leaflets. The membrane thickness exhibits a gradual decrease of nearly 6%, from an initial value of 3.8 to 3.6 nm by ≈ 600 ns, corresponding to the stage during which caffeine molecules have already fully penetrated the membrane and the stacking gradually disperses. Subsequently, the bilayer thickness increases and eventually reaches a plateau value of ≈3.8 nm, which is comparable to the thickness for a caffeine-free DPPC system under the same thermodynamic conditions and molecular model, as reported in our previous study [43]. The reduction in bilayer thickness can be attributed to the transient phase of the simulation, during which caffeine aggregates, penetrates and subsequently disperses in the membrane. In an incompressible bilayer, lateral area expansion is usually accommodated by a decrease in thickness so that the overall membrane volume remains effectively constant. However, in the present system, the bilayer thickness is almost unaffected after the complete dispersion of caffeine in the membrane, indicating that caffeine permeation has only a subtle effect on the overall bilayer thickness.

A previous study combining XRD and MD simulations [38] reported an increase of ≈ 2.6% in the bilayer thickness of a POPC membrane upon caffeine penetration. This increase was attributed to a reduction in the gauche fraction of lipid tails caused by the formation of water pockets near the headgroup region, as demonstrated through combined computational and experimental approaches. It is worth noting, however, that the study in [38] involved a different lipid composition, albeit of the PC class, and a much lower caffeine molar fraction (≈3%), complicating direct comparison with the present system. In addition, there are a few reports where amphiphilic helical peptides elicit the opposite behavior, resulting in a net increase in bilayer thickness [59]. In parallel, a membrane-thinning effect was reported in the experimental study of a preloaded supported POPC bilayer [39]. Their methodology differs from that of the present simulations, as an equilibrated bilayer was subsequently exposed to caffeine in bulk water, allowing the system to relax toward an interfacial partitioning state over time.

To further quantify the area per lipid (APL), we use the Voronoi tessellation method to handle the non-uniform packing of lipids due to the presence of caffeine molecules. It is further employed to calculate the area per caffeine (APC). The positions of the phosphorus atoms of the lipid headgroups (shown as black circles) and of the centers of mass (C.o.M.s) of caffeine molecules (shown as green asterisks) are projected in each leaflet onto the *x*–*y* plane under periodic boundary conditions following a similar approach to Shinoda et al. [60]. Effectively, a 2D Voronoi diagram is constructed using the Voronoi routine of SciPy (scipy.spatial.Voronoi) through the MDAnalysis (version 2.9.0) modelling tool [61,62]. For a given configuration, all P atoms and the C.o.M.s of caffeine molecules are assigned to the top or bottom leaflet based on the closest proximity of their *z*-coordinate. For each leaflet, the Voronoi construction yields one polygon (cell) and associated area for every DPPC molecule (blue for top leaflet and red for bottom leaflet, respectively) and caffeine molecule (yellow). Each of the cells corresponds to the area of the corresponding molecule. The images are produced using Matplotlib (version 3.10.3) [63] and Python (version 3.10) [64].

**Figure 8:**
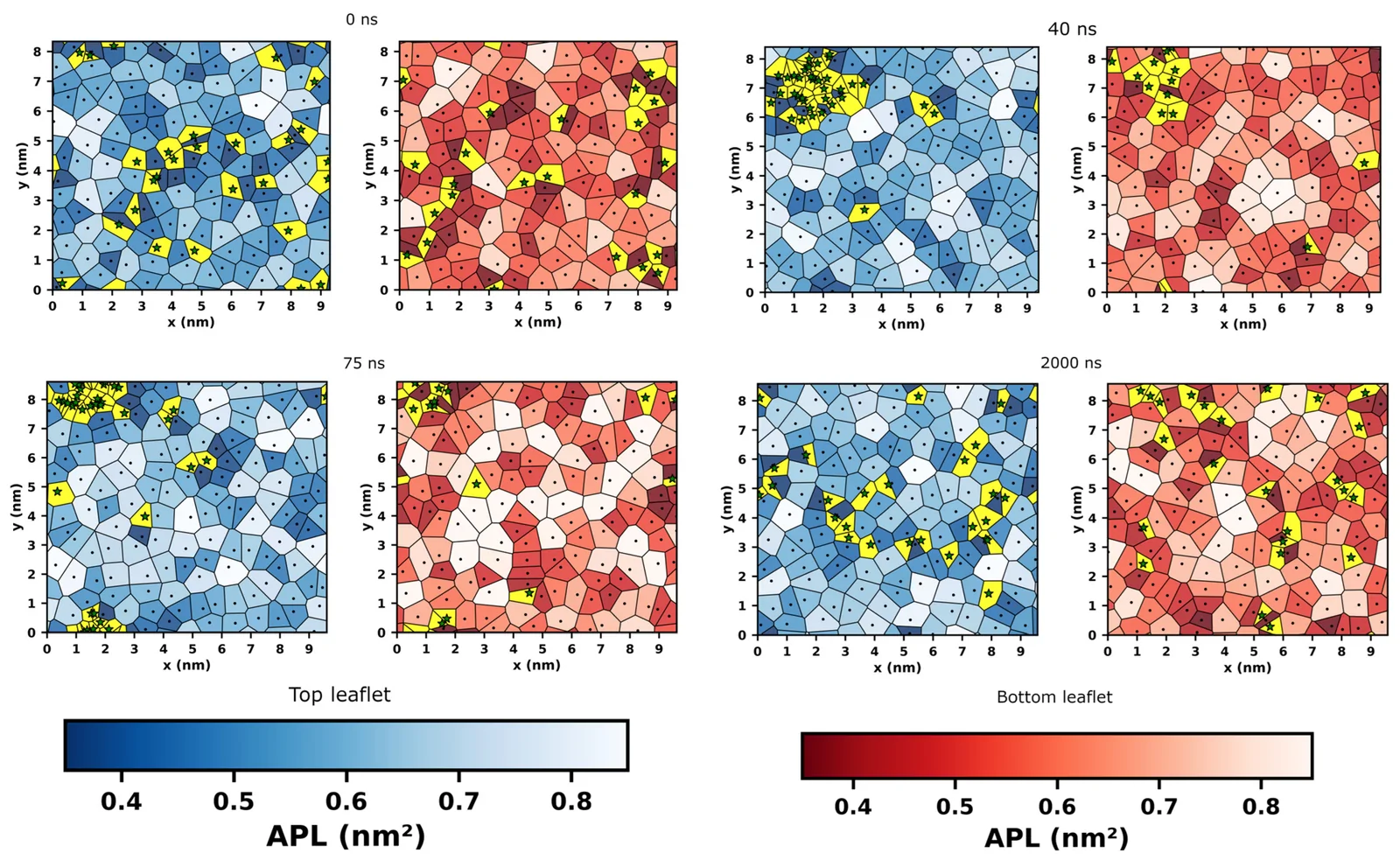
Voronoi diagrams for the top (**left** side, blue color) and bottom (**right** side, red color) leaflets at different time instances: *t* = 0 (energy minimization), 40, 75, and 2000 ns. The color bar for the top and bottom leaflets is shown in blue and red shades, respectively. Projected coordinates of the phosphorus (P) atoms and centers of mass (C.o.M.s) for the caffeine molecules are shown as black circles and green stars, respectively. The Voronoi polygon around each caffeine molecule is shown in yellow. Each DPPC and caffeine molecule is assigned to either the top or bottom leaflet based on the closest proximity according to its *z*-coordinate. The darker the Voronoi cell (polygon), the denser the local environment.

The panels in Figure 8 show the Voronoi cell distribution for both leaflets at selected time steps. The darker the color of the Voronoi polygon around the lipid, the denser the local environment and the lower the APL. A color scale is provided, showing the APL variation with the color shades. The Voronoi tessellation in the top left panel of Figure 8, corresponding to the energy-minimized structure, shows the initial caffeine dispersion across the surface of the membrane. The highly concentrated caffeine population at 40 ns (top right panel of Figure 8), is strongly related to the caffeine aggregation, as shown in Figure 4a,b, respectively. The concentrated caffeine domain shrinks slightly due to the columnar stack at 75 ns (bottom left), as also seen in Figure 4c. Finally, the Voronoi diagram in Figure 8 (bottom right) at 2000 ns shows a dispersed caffeine distribution in accordance with the system snapshots, as shown in Figure 4d.

**Figure 9:**
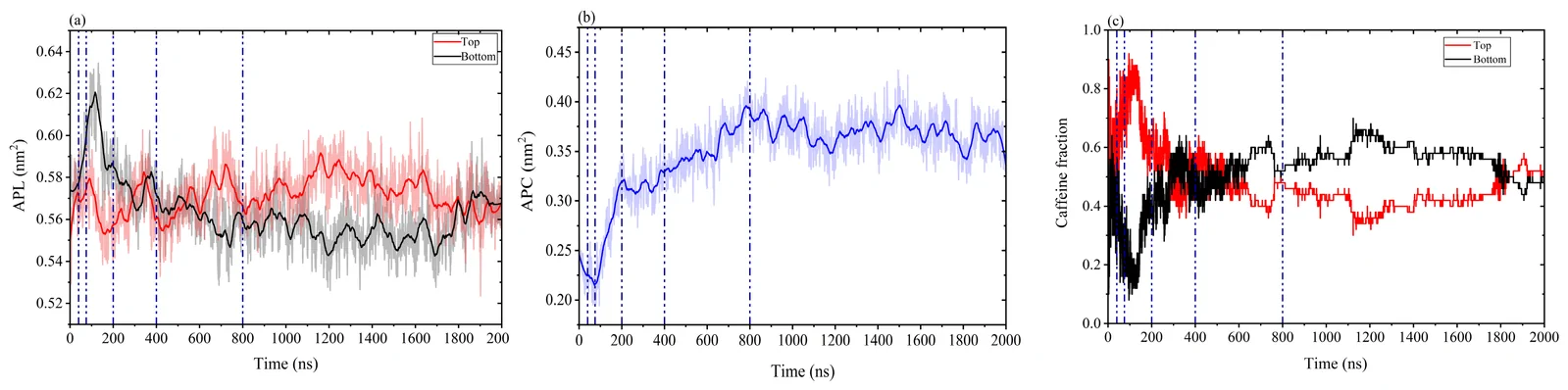
(**a**) Area per lipid (APL) for the top (red color) and bottom (black color) leaflets, and (**b**) area per caffeine (APC, blue color) as a function of time. Thin and thick lines represent the instantaneous and running average values, respectively. (**c**) Caffeine fraction in the top (red) and bottom (black) leaflets as a function of time. The blue dot-dashed vertical lines are placed at *t* = 40, 75, 200, 400 and 800 ns to indicate the different phases of the caffeine permeation process.

For each configuration, the average APL, calculated separately for the top and bottom leaflets, and the average APC are calculated, as seen in Figure 9a,b, respectively. The APC shows a slight drop at early times (*t* ≤ 70 ns), followed by an appreciable increase up to *t* ≈ 200 ns, succeeded by a more gradual raise until 700–800 ns. This behavior is closely correlated with initial aggregation, stacking and the progressive dispersion of the caffeine clusters, indicating that the breakup of the aggregates contributes to the increase in membrane surface area.

The APL in the top leaflet remains stable, within thermal fluctuations, throughout the simulation, whereas the lower leaflet shows sharp increase during the first 100 ns, followed by a gradual decrease to a plateau value of ~0.55 nm^2^ by ~600 ns. This asymmetric behavior arises from the non-uniform caffeine distribution in both leaflets, as shown in Figure 9c. This trend can be understood if we consider that the caffeine self-organization (clustering followed by stacking) and eventual penetration take place through just one of the two (the top) leaflets of the bilayer, as clearly seen in Figure 2b,c. As a result, the bottom leaflet contains fewer caffeine molecules, allowing lipids to occupy a larger effective surface area and causing the observed initial increase in APL. As the caffeine aggregates disperse and the caffeine population becomes more symmetrically distributed between the two leaflets by 400 ns, the area per lipid in both leaflets gradually converges to similar values.

#### 2.6.2. Deuterium Order Parameter

**Figure 10:**
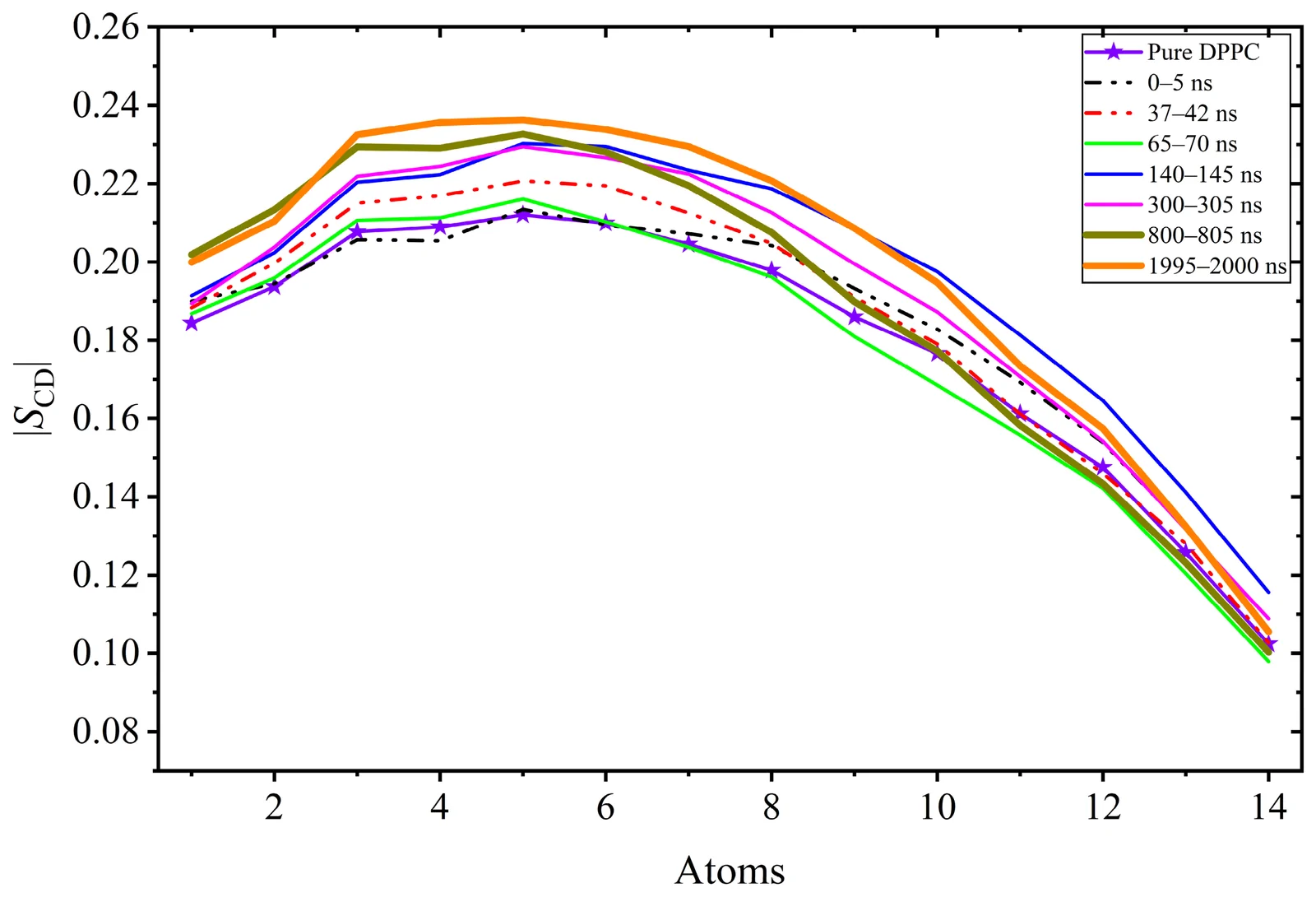
Deuterium order parameter, |*S_CD_*|, for the sn-2 chain of the DPPC membrane at various time intervals. Also shown are the corresponding data for the caffeine-free (pure) DPPC system.

Phospholipids with two distinct acyl chains, labeled sn-1 and sn-2, are bonded to a glycerol backbone, as seen in Figure 1a. Typically, the acyl chain at the sn-1 position is saturated, while the chain at the sn-2 position is unsaturated and contains a different number of carbon atoms than the sn-1 segment [65,66]. Interaction of foreign molecules with a lipid bilayer can alter acyl chain alignment and thereby perturb membrane structure. To quantify the effect of the caffeine on the chain ordering, the deuterium order parameter [67], ${|S}_{CD}|$ is calculated using the formula ${|S}_{CD}|=\frac{1}{2}\langle 3{cos}^{2}\alpha_{C-H}-1\rangle ,$ where $\alpha_{C-H}$ is defined as the angle between the C–H bond vector and the bilayer normal (aligned with the *z*-axis in the present simulations), as shown in Figure 10.

The changes in the $|S_{CD}|$ order parameter for sn-2, to be discussed below, are evaluated relative to a caffeine-free membrane, as reported in [43]. During the 37–42 ns interval, $|S_{CD}|$ near the headgroup (low atom indices) exhibits deviations with respect to the caffeine-free system, while no change is detected near the terminal tail region (high atom indices). In this interval, caffeine initiates the permeation process into the membrane, through the clustering and stacking phases. Accordingly, some molecules lie adjacent to the headgroup region, leading to this pattern in the *S_CD_* order parameter. For 140–145 ns, caffeine molecules, forming a large aggregate in the upper leaflet, have already penetrated the interface lying in the hydrophobic region, as indicated by the cluster analysis and the representative snapshots in the earlier sections. The corresponding effect on the order parameter is therefore prominent in both the headgroup and acyl chain segments. The same pattern is also observed for 600–605 ns, where the caffeine cluster seems to expand to both leaflets and starts to disperse, as indicated by Figure 2f.

The enhancement is attributed to the presence of caffeine molecules not only near their preferred location at the headgroup–acyl chain (HG–AC) interface but also transiently within the central region of the bilayer, as evidenced by the mass density and caffeine fraction profiles (Figure 3b,c). At a later interval (800–805 ns), a pronounced increase in $|S_{CD}|$ is observed near the acyl chain nearer to the headgroup region, reaching approximately 10–12% for the fourth carbon atom by 1995–2000 ns, with minimal effect on the terminal atoms.

Overall, these results indicate that the localization of caffeine at the headgroup–tail interface during the equilibration phase increases the order parameter in the upper segments of the acyl chains, consequently reducing local fluidity in these regions and potentially causing altered membrane function [68,69]. A similar trend is observed for the sn-1 chain (Figure S2), further supporting this interpretation.

### 2.7. Effect of Initial Caffeine Placement

**Figure 11:**
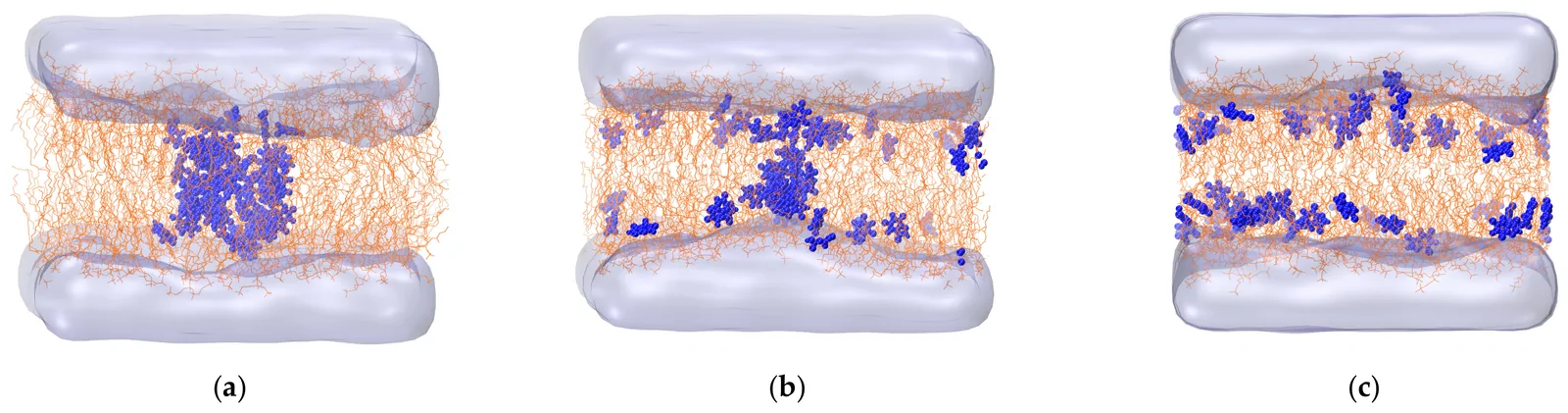
System configurations (snapshots) for the modeling setup where 50 caffeine molecules are initially placed, in the form of a single droplet, in the hydrophobic phase of the DPPC membrane. (**a**) Initial state at *t* = 1 ns, (**b**) *t* = 500 ns and (**c**) *t* = 2000 ns (final state). Color coding and representation follows that of Figure 1.

The influence of the initial caffeine placement on its dispersion and spatial distribution inside the membrane is assessed by comparing the primary modelling approach against an alternative setup where caffeine molecules, corresponding to the same concentration, are placed, in the form of a droplet, inside the membrane (hydrophobic phase of the lipid), keeping the rest of the simulation parameters the same (2000 ns of NPT, *T* = 323 K and *P* = 1 bar). Representative snapshots illustrate the initial, intermediate, and final states, as seen in the Figure 11a, b, and c, respectively. Visual inspection (compare, for example, Figure 11c with Figure 2g) demonstrates that in the final phase, the caffeine molecules adopt positions that very closely follow the ones when caffeine is placed in the aqueous environment.

**Figure 12:**
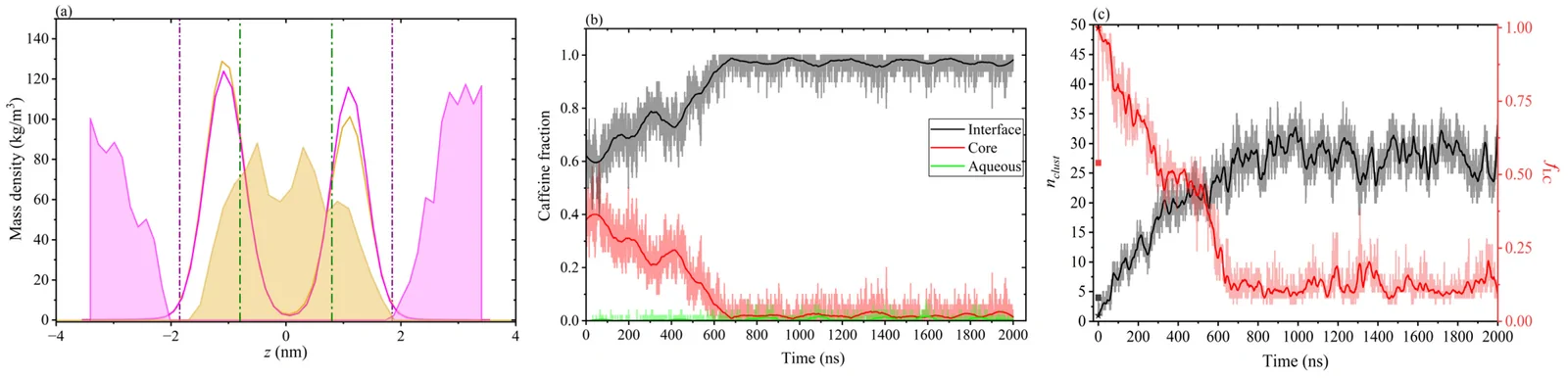
(**a**) Mass density profile for the caffeine molecules at the beginning (filled curve) and ending (open curve) of the simulation for the two different placement patterns: in water (magenta color) and in the hydrophobic core of the DPPC membrane (orange color). Vertical black and blue dashed-dotted lines mark the peaks of the HG and AC mass density profiles, respectively, as identified in Figure 3a. (**b**) Caffeine fraction as a function of time in aqueous, interfacial and core regions, as identified in Figure 3c, when caffeine molecules are initially placed in the form of a droplet in the DPPC core. (**c**) Number of distinct clusters (black line, left *y*-axis) and caffeine fraction in the largest cluster (red line, right *y*-axis) as a function of time. Thin and thick lines correspond to instantaneous and running average values, respectively. Asterisks and square symbols denote the cluster data as extracted from the system configuration after energy minimization and the 1 ns-long equilibration NVT MD simulation, respectively.

The caffeine spatial positioning, corresponding to equilibrium, remains unchanged across the two placement patterns (in the water domain and in the hydrophobic core of DPPC), as evidenced by the mass density profiles presented in Figure 12a. The comparison demonstrates that caffeine resides consistently at the headgroup–tail interface, regardless of the initial positioning. This can also be confirmed from the time-dependent caffeine fraction plot for various distances along the membrane normal, as seen in Figure 12b. After 700 ns, the caffeine molecules lie in the range of 0.5 < |z| ≤ 2 nm, i.e., adjacent to the headgroups for |z| ≤ 0.5 nm. The final caffeine partitioning when the molecules are placed in the DPPC hydrophobic phase (Figure 12b) is thus the same as when the molecules are dispersed in the aqueous environment (Figure 3c).

The cluster analysis data, as seen in Figure 12c, clearly demonstrates that the caffeine fraction in the largest cluster after energy minimization is the maximum possible, as all molecules are jointly placed in the form of a droplet inside the hydrophobic phase. This fraction is significantly reduced to a value of 0.54 after the 1 ns-long NVT equilibration. The number of distinct clusters gradually increases with time when the initial droplet starts disintegrating until it reaches the equilibrium value at around 700 ns.

**Figure 13:**
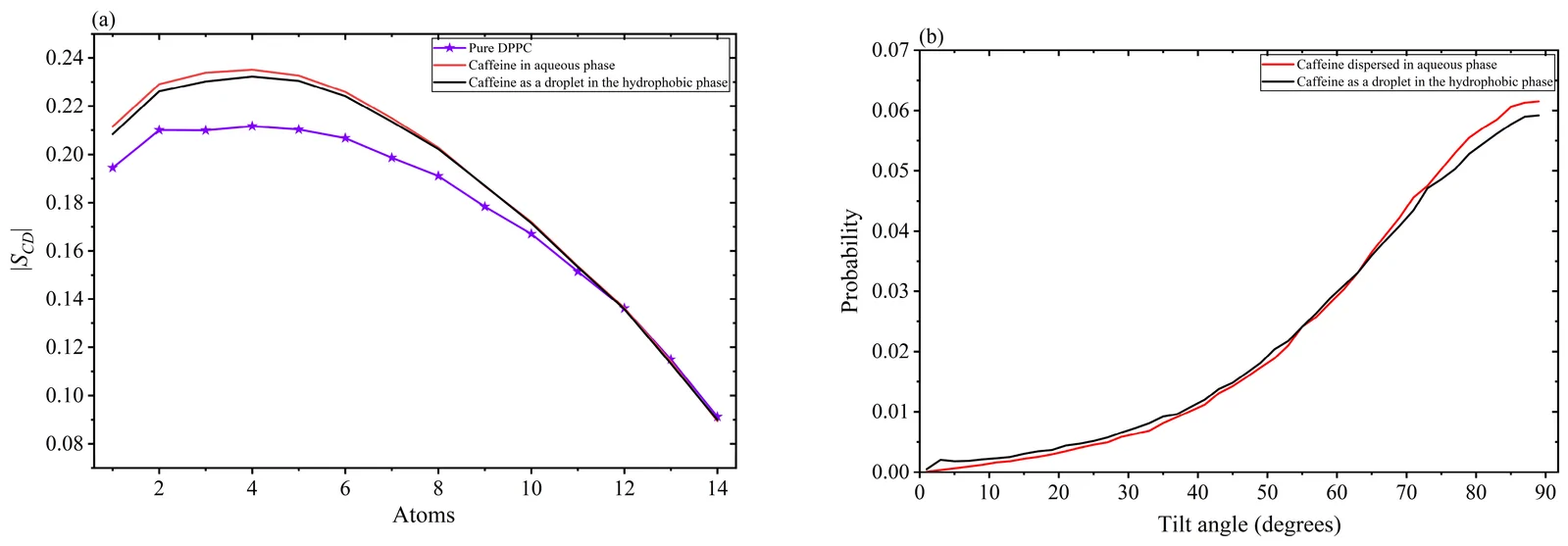
(**a**) Deuterium order parameter (sn-1) at the end of the simulation for the pure DPPC system (violet line with stars), and for the caffeine-loaded ones, when caffeine molecules are dispersed in the aqueous phase (red curve) and as a droplet in the hydrophobic phase of the DPPC (black curve). (**b**) Distribution of the caffeine tilt angle at the end of the simulation for the two different placement schemes.

In parallel, when caffeine is placed as a droplet in the DPPC hydrophobic phase, its dispersion consistently elevates the deuterium order parameter (Figure 13a) in an almost identical way as the placement in the aqueous domain. Similarly, the placement pattern has no appreciable effect on the preferential alignment of the caffeine molecules parallel to the acyl chains, as demonstrated by the data of Figure 13b.

### 2.8. Potential of Mean Force and Caffeine Translocation

**Figure 14:**
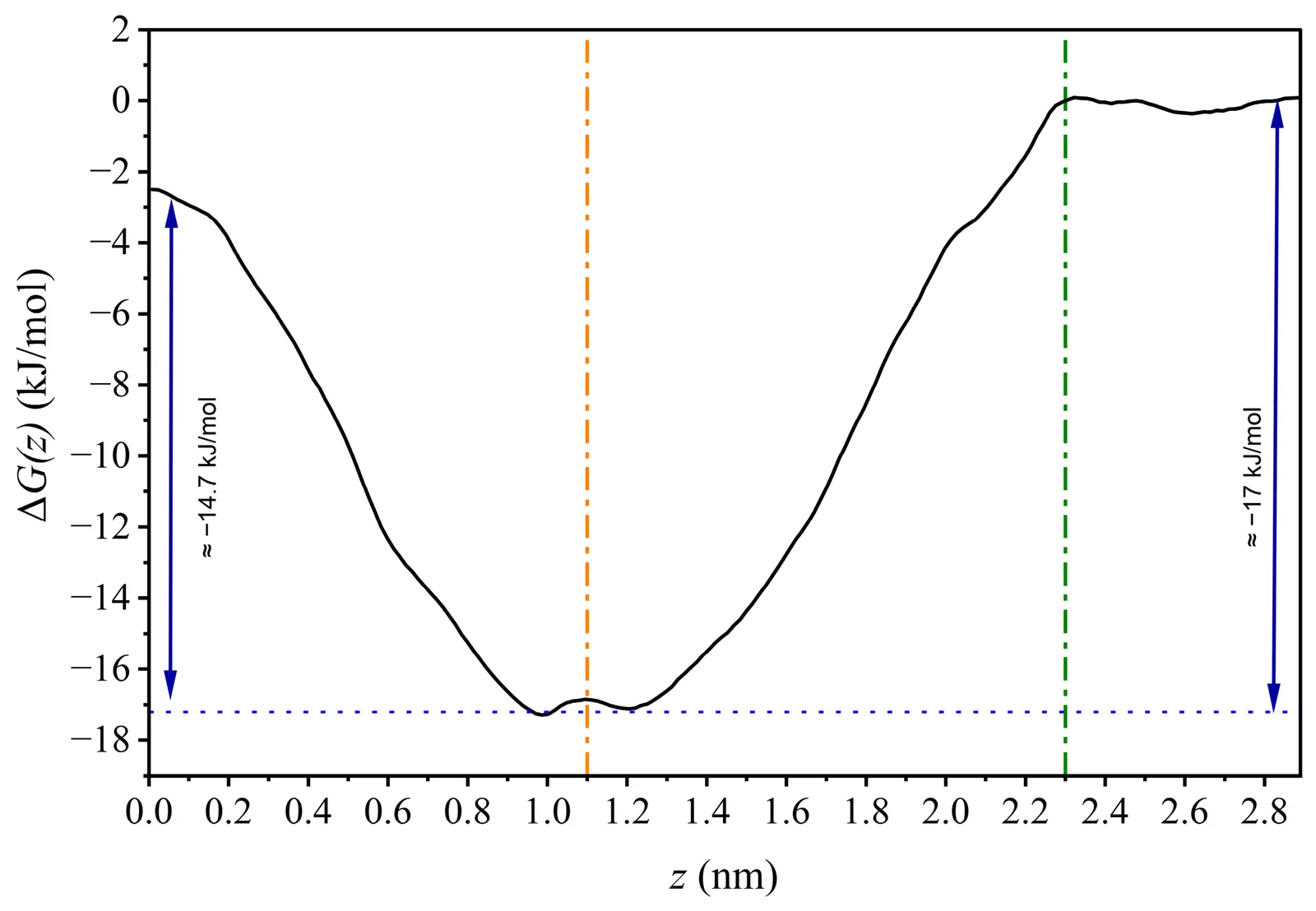
The PMF profile representing the relative free energy profile, $∆G\left(z\right)$, for caffeine as a function of the *z* coordinate, when it is pulled from the center of the membrane to the water layer. The local minimum at *z* ≈1.0 nm and the onset of the water layer at *z* ≈2.3 nm are shown by orange and green dot-dashed lines, respectively.

We calculate the potential of mean force (PMF) of a caffeine molecule along the bilayer normal. The caffeine molecule is initially placed at the hydrophobic core (z=0) of the DPPC membrane and is then pulled along the *z*-axis to the water layer through one of the two leaflets. Thus, PMF represents the relative free energy landscape, $∆G\left(z\right)$, experienced by the caffeine along the bilayer normal, as shown in Figure 14. $∆G\left(z\right)$ is set to zero in the bulk water phase.

Furthermore, the membrane/water partition coefficient, *P*, is determined using the following equation [36,37,70]:

(1) $$P_{memb/water}=\frac{2}{h}\int_{0}^{h}e^{-∆G\left(z\right)/k_{B}T}dz$$

where *k*_B_ and *T* are the Boltzmann constant and the absolute temperature, respectively, and *h* is the half bilayer thickness. *h* is determined from the free energy profile $∆G\left(z\right)$ as the distance from the bilayer center to the point where free energy reaches a plateau in the bulk water regime, which is practically zero.

The PMF profile in Figure 14 shows a deep free energy minimum (≈−17 kJ/mol) at z ≈ 1.0–1.2 nm, corresponding to the HG-AC interface located inside DPPC membrane. The free energy at the membrane center (*z* = 0) is lying slightly below that of bulk water. The large free-energy gaps of approximately −14.7 and −17 kJ/mol from the hydrophobic core and bulk water, respectively, to the free energy minimum clearly explain the reason for caffeine migration towards the HG–AC interface irrespective of its initial placement (bulk water or hydrophobic core).

In a previous simulation study involving caffeine and DOPC lipids, the free energy minimum is located inside the membrane, which is similar to our observation [36]. The partition coefficient $P_{memb/water}$ and log *P* are found to be ≈336 and +2.5, respectively, predicting a high affinity of the caffeine molecules to the DPPC membrane, which is consistent with the present simulation results.

The mass density profile (Figure 3b) indicates a very low but finite caffeine presence at the hydrophobic core. Additionally, the fluctuations in caffeine fraction (Figure 9c) in the top and bottom leaflets in the equilibration phase (800–2000 ns) identify very limited caffeine translocation. For quantitative estimation, we additionally calculate the inter-leaflet transition of caffeine molecules through the hydrophobic core, as seen in Figure S3 (Supplementary Materials).

### 2.9. System Size Effect and Reproducibility

We also simulate a smaller system containing 128 DPPC and 25 caffeine molecules to study the system size effect while keeping all other simulation conditions unchanged. A smaller system is chosen instead of a larger one due to the significantly higher computational cost of modelling larger systems and the need to access simulation times in the order of microseconds. The smaller system shows similar behavior, including aggregation, stacking, penetration, and gradual dispersion, as seen in Figure S4 (Supplementary Materials). The final mass density profile, orientation of caffeine, and deuterium order parameter of the DPPC also remain similar to those of the larger system (Supplementary Materials Figures S4–S6). However, as the total number of caffeine molecules is half compared to the original system, the cluster size decreases accordingly, consequently leading to a rapid dispersion phase.

To examine reproducibility, we also perform additional independent simulations (256 DPPC and 50 caffeine molecules) with random initial velocities for both schemes regarding the initial caffeine placement. For the insertion pattern inside the membrane, we conduct two independent simulations where caffeine molecules are placed as a cluster and scattered in the hydrophobic phase. The corresponding results, provided in the Supplementary Materials (Figures S4–S7), show no significant differences between the independent simulations. The cluster analysis, mass density profile, caffeine orientation, and deuterium order parameter of the DPPC show excellent agreement across the independent systems, demonstrating the reproducibility of the simulations.

## 3. Materials and Methods

All MD simulations are performed using the open-source software suite Gromacs (version 2021.4) [71] employing the Berger lipid (L-B) force field [72], an interaction potential widely used in the last two decades for the simulation of lipid-based systems [36,43,73,74,75,76,77,78,79,80,81]. The L-B is a united-atom (UA) interaction potential based on the GROMOS 54a7 force field [82]. In the UA description, the CH, CH_2_ and CH_3_ units are treated as single interaction sites, and the corresponding hydrogens are not explicitly present in the model description. Owing to this grouping, the UA representation is nearly three times faster than the all-atom (AA) representation, as shown in a recent publication involving pure DPPC membrane [43], offering significant computational efficiency and enabling expanded simulation times. At the same time, it retains more explicit information compared to coarse-grained models [83,84,85], allowing for a balance between accuracy and efficiency [43,86,87,88]. However, it is important to state that for complex biological processes, a detailed comparison with available experimental data and past simulation studies is required to assess the predictive power and validity of each employed force field.

The Visual Molecular Dynamics (VMD) [89] software (version 1.9.3) is used for all visualizations of the computer-generated system configurations. A bilayer membrane comprising 128 DPPC in each leaflet is created by replicating an initially equilibrated 64–64 membrane configuration in the bilayer plane [90]. Thirty water molecules per DPPC are added, ensuring full hydration [91]. The simple point charge (SPC) model [92] is used for the water representation. All bonds are constrained to their equilibrium lengths with the linear constraint solver (LINCS) [93] algorithm. The bilayer membrane is set in the *x*–*y* plane, and consequently the bilayer normal aligns to the *z* axis. Periodic boundary conditions are applied on all dimensions, and a cut-off distance of 1.2 nm is implemented for the Lennard–Jones interactions and the real space part of electrostatic ones. The particle-mesh Ewald (PME) method [94] is used to compute long-range electrostatic interactions. For energy minimization, the steepest descent algorithm is implemented.

All production MD simulations are performed in the isothermal–isobaric (NPT) ensemble with Nose–Hoover thermostat [95,96,97] coupling to 323 K; constant lateral pressure along the *x*–*y* plane is maintained using semi-isotropic Parrinello–Rahman pressure [98,99] coupling to 1 bar. The pressure along the *z* axis is also kept at 1 bar. The integration time step is set to 2.0 fs, and system configurations (frames) are saved every 100 ps.

Caffeine representation and topology are performed by using the Automated Force Field Topology Builder (ATB), computing partial charges using quantum mechanical optimization at the B3LYP/6-31G* level of theory [100,101,102]. In the second step, caffeine molecules are inserted into the corresponding DPPC + water system. It is well known that caffeine, when dispersed in aqueous solutions, forms stacks [41,42]. As stated in the Results section, two different insertion patterns are followed, with the caffeine molecules being randomly dispersed in the aqueous phase and in the form of a droplet in the hydrophobic core of the membrane.

In the present study, caffeine concentration is chosen as 20% mol, ensuring molecular organization in the form of clustering and stacking, as we demonstrate later. Practically, this corresponds to the insertion of 50 caffeine molecules. Accordingly, the final system is composed of 256 fully hydrated molecules of DDPC and 50 molecules of caffeine.

We initially conduct NPT MD simulations with a total duration of 200 ns on the pure-DPPC-plus-water system at *T* = 323 K and *P* = 1 bar. After the caffeine molecules are inserted into the system, an energy minimization is applied, followed by an NVT MD simulation of 1 ns (*T* = 323 K). The production NPT MD phase corresponds to a total simulation time of 2000 ns (2 μs), resulting in a trajectory of 20,000 frames (system configurations).

For the Potential of Mean Force (PMF) calculations for the caffeine–membrane interactions, we follow a methodology like that of [36], where umbrella sampling simulations are performed along the membrane normal (*z*-axis). The reaction coordinate is defined with respect to the center of the simulation cell (*z* = 0), which coincides with the center of the DPPC lipid bilayer and the *z* coordinate of the center of mass (C.o.M.) of the caffeine molecule. The sampling pathway is discretized into 45 independent bins, equidistantly spaced at intervals of 0.064 nm, spanning from the bilayer core (*z* = 0 nm) to the bulk aqueous phase (*z* = 2.88 nm). Each bin is simulated for a total of 30 ns. These windows are subsequently analyzed using the Weighted Histogram Analysis Method (WHAM), as implemented in Gromacs (*gmx wham* module). In the analysis, only the last 20 ns are considered for equilibration purposes.

Two different patterns for initial caffeine distribution are used: in the first one, the caffeine molecules are inserted into the water volume of the system, while in the second, they are directly inserted into the hydrophobic part of the lipid membrane. Our manuscript primarily focuses on the results of the first scheme, where the caffeine molecules are inserted in the aqueous solution, while the second placement scheme is briefly discussed for comparison.

## 4. Conclusions

This study investigates the penetration process of caffeine in a solvated DPPC bilayer using united atom MD (2 µs) simulations. Caffeine molecules initially placed in an aqueous solution tend to form transient aggregates that rapidly evolve into ordered stacks and partition into the membrane by roughly 200 ns. The nematic correlation function successfully captures the multilayer stacking, and the interplanar spacing between the caffeine planes are similar to those in aqueous and NaCl salt solutions [51,53,56]. During partitioning, the caffeine stack gradually fragments and reaches full dispersion by 800 ns. The dispersed phase remains stable throughout the simulation, and the caffeine molecules are located near the headgroup–acyl chain interface and are preferentially oriented parallel to the acyl chains. This caffeine location and orientation are in good agreement with the previous simulation [36,37] and experimental results [38] for phosphatidylcholine lipids. We also carried out another simulation by initially placing the caffeine molecules in the hydrophobic core of the membrane, which eventually disperse and transit to the same equilibrium position (near the headgroup–acyl chain interface). The caffeine mass density profile and caffeine fraction show that the molecules very infrequently cross the hydrophobic core, suggesting that direct, inter-leaflet translocation through the membrane is unlikely.

The process of caffeine permeation temporarily reduces the bilayer thickness, which recovers on equilibration. Concurrently, the surface area of the membrane swells up by ≈8% to accommodate the caffeine molecules. The presence of caffeine molecules causes membrane stiffening, as evident by the increase in acyl chain ordering (characterized by the *S_CD_* order parameter) near the hydrophilic part of the membrane, while the hydrophobic part remains unperturbed.

The relative free-energy profile, ∆G(z), exhibits a well-defined minimum near the HG–AC interface, explaining the preferential accumulation of caffeine there, irrespective of its initial placement in the bulk water or the hydrophobic membrane core. Caffeine transition from the membrane into the bulk water is strongly restricted by a large barrier of ~17 kJ mol^−1^, and the translocation of caffeine through the core is a rare event, due to a barrier of −14.7 kJ mol^−1^ separating the hydrophobic core with the preferred HG–AC interface. Furthermore, the high positive logP value of 2.5 for the membrane–water system suggests a strong affinity of caffeine for the membrane.

The preferential location of caffeine molecules near the HG–AC interface is in qualitative agreement with the binding site predicted for selective antagonist ZM241385 [103] and for caffeine [9]. We note that, for both these antagonists, the primary binding site on A2A receptors is found to be inside the transmembrane region, though facilitated by an intermediate binding site in the extracellular region of the cell. However, the present simulations do not include an explicit receptor environment and therefore do not address ligand–receptor binding or kinetics. Consequently, any connection between membrane localization and receptor interaction remains speculative and warrants further investigation using explicit receptor–membrane simulation models.

The established trends are reproducible, as verified by conducting simulations on a smaller system and through two independent simulations differing in the initial velocities and placements of the caffeine molecules.

We should note that the present simulation findings, including the relative free-energy profile and consequently the logP, may depend on the force field employed. A detailed comparison of force fields commonly employed on caffeine–membrane interactions will be addressed as part of future studies.

This contribution could aid in the development of membrane-based drug delivery systems by providing a detailed molecular understanding of caffeine–DPPC interactions and on caffeine’s impact on bilayer structure and dynamics.

## Data Availability

All raw and processed data from the simulations reported here are fully available upon request.

## Supplementary Materials

The following supporting information can be downloaded at: https://www.mdpi.com/article/10.3390/ijms27177704/s1.

## Abbreviations

The following abbreviations are used in this manuscript:

DPPC	1,2-dipalmitoyl-sn-glycero-3-phosphocholine
MD	molecular dynamics
CNS	central nervous system
HCC	hepatocellular carcinoma
CKD	chronic kidney disease
BLF	glycoprotein bovine lactoferrin
DODAB	dioctadecyldimethylammonium bromide
QENS	quasielastic neutron scattering
ADME	absorption, distribution, metabolism, and excretion
POPG	palmitoyl-2-oleoylphosphatidylglycerol
DOPC	1,2-dioleoyl-sn-glycero-3-phosphocholine
PMF	potential of mean force
POPC	1-palmitoyl-2-oleoyl-sn-glycero-3-phosphocholine
ATB	Automated Force Field Topology Builder
VMD	Visual Molecular Dynamics
PME	particle-mesh Ewald
PC	phosphatidylcholine
C.o.M.	Center of mass
HG-AC	headgroup–acyl chain
DFT	Density Functional Theory
APL	area per lipid
S_CD_	deuterium order parameter
