# Supplementary material for: Molecular Insights into Caffeine Stacking, Partitioning, and Organization in DPPC Lipid Bilayers from Microseconds-Long Molecular Dynamics Simulations
Source: Int J Mol Sci. 2026 Aug 28;27(17):7704. doi: 10.3390/ijms27177704 (PMC13565828; doi:10.3390/ijms27177704)
Supplement: Supplementary file 1 [file ijms-27-07704-s001.zip › ijms-4472621-supplementary.pdf]

Supplementary Materials: Caffeine Stacking, Partitioning, and Organization in a Lipid Bilayer from Microsecond-Long Molecular Dynamics Simulations

Subhalaxmi Das[1], Nikos Ch. Karayiannis[2], Supriya Roy[1,*]

1 School of Applied Sciences, KIIT Deemed to be University, Bhubaneswar, Khurda, Odisha-751024, India

2 Institute for Optoelectronic Systems and Microtechnology (ISOM) and Escuela Técnica Superior de Ingenieros Industriales (ETSII), Universidad Politécnica de Madrid (UPM), José Gutiérrez Abascal 2, E-28006 Madrid, Spain

* Corresponding author, e-mail: *supriyophy163@gmail.com*

Cluster Analysis

Figure S1 presents the same system snapshots as Figure 4 of the main manuscript but through a different, more detailed representation.

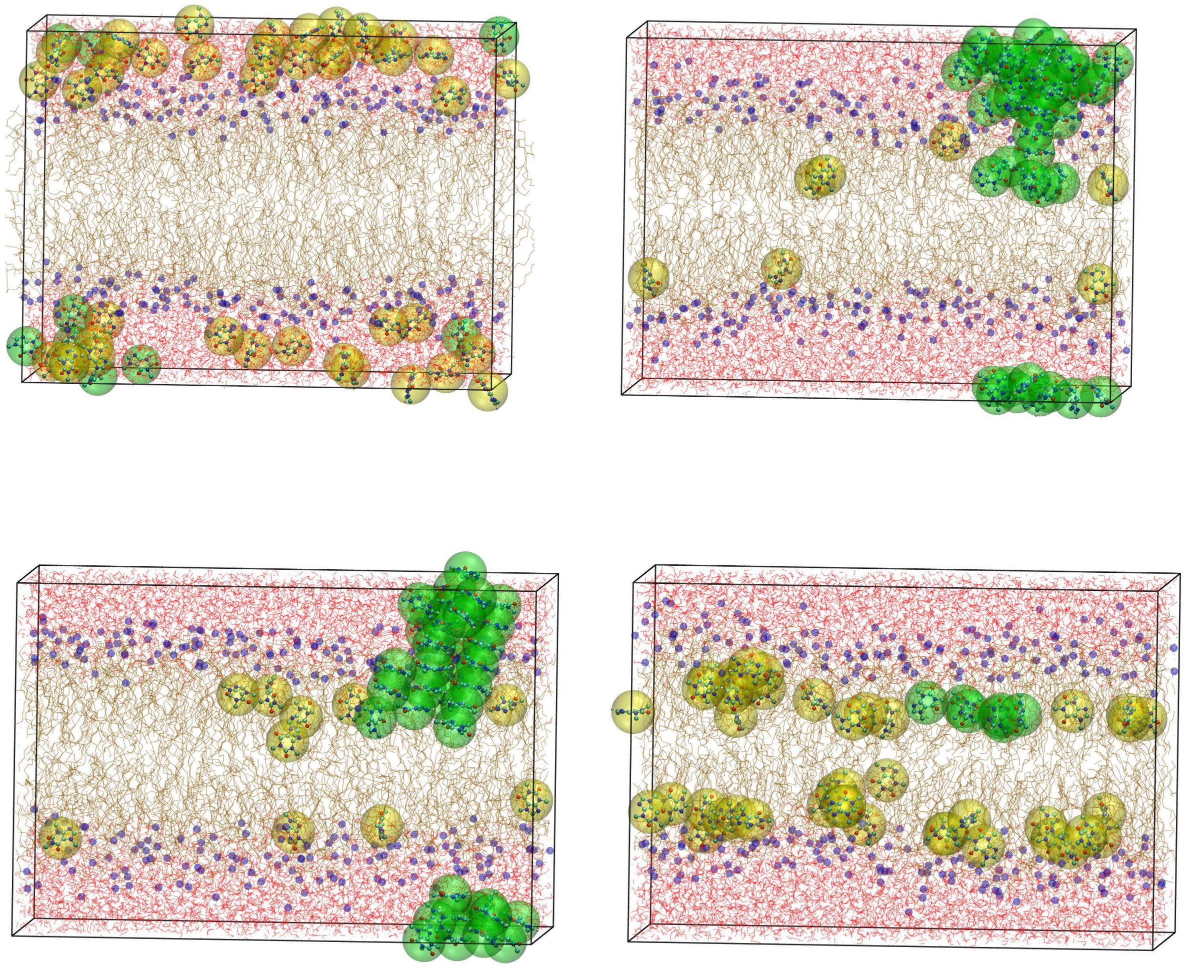

Figure S1. System snapshots at t = 0 (top left), 40 (top right), 75 (bottom left) and 2000 ns (bottom right), as obtained from the cluster analysis. Water and DPPC molecules are shown as red and brown lines, respectively. Blue spheres correspond to the nitrogen atoms of the DPPC headgroups. Caffeine molecules are shown in a CPK representation. Transparent spheres around the center of mass of caffeine molecules have size equal to the radius implemented for cluster detection (1 nm). Green indicates caffeine molecules belonging to the largest cluster and yellow corresponds to caffeine molecules in smaller clusters.

Deuterium order parameter

With respect to the deuterium order parameter and its evolution during the caffeine permeation process the sn-1 chain segment shows the same behavior, as seen in Figure S2, as the sn-2 chain one (Figure 10 in the main text).

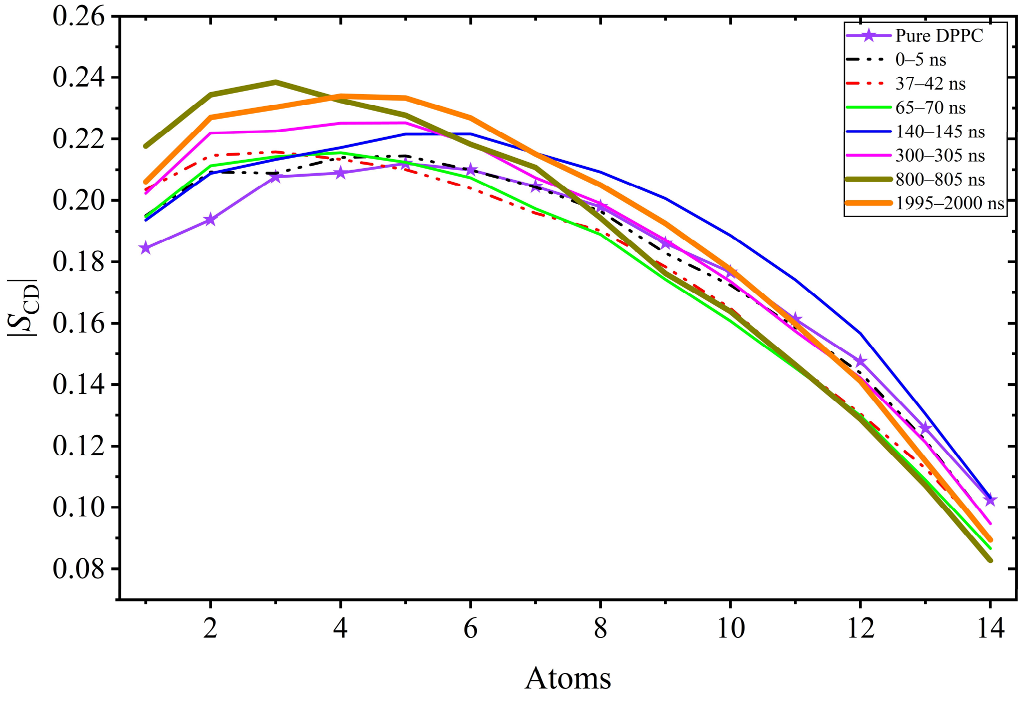

Figure S2. Deuterium order parameter for the sn-1 chain segment, |S_{CD}|. Different colors correspond to different time intervals as indicated in the label. The purple line with stars corresponds to the order parameter for the pure DPPC system under the same hydration conditions.

Caffeine translocation between leaflets

Caffeine translocation between the two membrane leaflets is calculated using the MDanalysis[1], [2] . At each simulation frame, the time-dependent physical boundaries of the membrane core are identified by calculating the mean z-coordinate of all DPPC phosphorus atoms. The independent mean positions of the upper, z_{upper} and lower leaflet, z_{lower} headgroups are calculated including the middle point of their distance, z_{mid}. A caffeine molecule is considered as been translocated when withing two successive frames (system configurations) its center of mass passes from one leaflet to the other (from top to bottom and vice versa) while it remains within the limits of the membrane core. Figure S3 shows the fraction of caffeine molecules transitioning from one leaflet to the other as a function of simulation time for the whole MD trajectory. The fraction of translocated caffeine molecules registers its maximum values during the 100 - 600 ns time interval, which corresponds to the redistribution of the caffeine molecules across the leaflets as the caffeine

cluster/stacking disperses. Leaflet translocation becomes significantly less frequent in the latter part of the simulation, dispersion phase near the HG in the membrane core, as indicated by the drop in the fraction (≈ 0.02 - 0.04).

The above trends are consistent with the caffeine mass density profile which shows a very small value at the hydrophobic core in the equilibration phase a seen in Figure 3(b) of the main manuscript. The translocation of caffeine is restricted evidently due to the high free energy barrier of -14.7 kJ/mol between the HG-AC interface and the bilayer core as further illustrated in the free-energy calculation (see Figure 14 of the main manuscript and corresponding discussion).

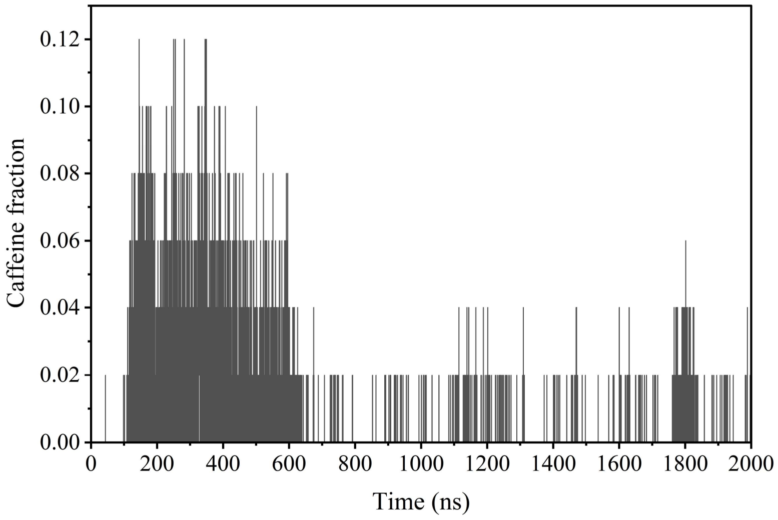

Figure S3. Fraction of caffeine molecules transitioning between the membrane leaflets versus time. Given the caffeine load implemented in the present simulation, a step of 0.02 shown above corresponds to 1 caffeine molecule.

Reproducibility and system size effect

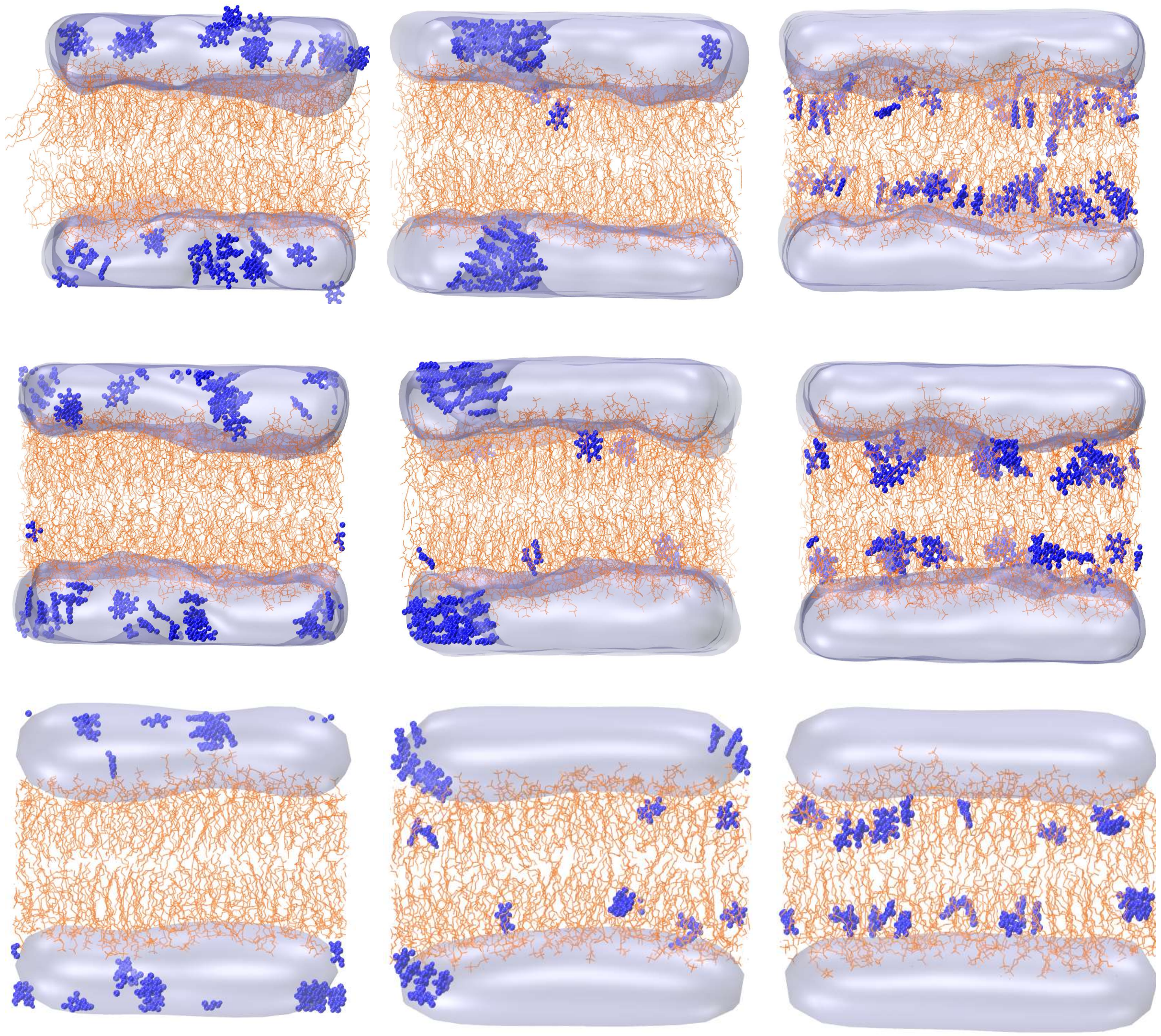

Figure S4. System snapshots at various time instances. From left to right: t = 0, 50 and 2000ns. Top and middle panels correspond to the independent simulations labeled as “sim_2” and “sim_3”, respectively. Bottom panels correspond to the smaller system realization with 128 DPPC molecules, labeled as “128DPPC-caf”. The water domain is represented here as a transparent surface, the DPPC molecules are shown as orange lines, and the atoms of the caffeine molecules are shown as blue spheres.

To study the reproducibility of the simulation findings and the effect of system size we conduct two additional independent simulations (referred to as “*sim*_2” and “*sim*_3” in the corresponding figure panels), by inserting the caffeine molecules to different (still random) positions in the aqueous volume and by assigning different initial velocities to each atom of the system. Additionally, a smaller system is constructed comprising 128 DPPC with 25 caffeine molecules (referred to as “128DPPC-caf”), maintaining

the same DPPC to caffeine ratio. The snapshots of all three systems are shown in Figure S4. In all independent simulations we observe identical phases in the caffeine permeation process including clustering, stacking, permeation and dispersion as discussed in the main text (which we refer here as "sim_1").

Figure S5 shows the results of the cluster analysis of all three systems. Qualitatively there appear no differences between the different systems and the main one.

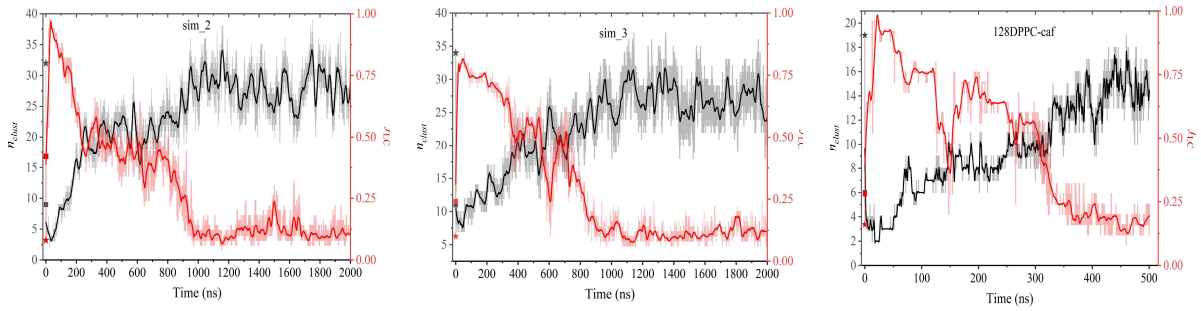

Figure S5. Cluster analysis for the two independent simulations ("sim_2" and "sim_3") and the smaller system ("128DPPC-caf"). Left y-axis (black curve) shows the number of clusters, n_{clust}, while right y-axis (red curve) shows the fraction of caffeine molecules in the largest cluster, f_{LC}. Thin and thick lines correspond to raw and smoothed values, respectively.

The caffeine permeates into the membrane through a process initiated by the formation of clusters, followed by ordered stacking, before reaching the headgroup-tail interface, as also confirmed by mass density profile of caffeine molecules in panel (a) of Figure S6.

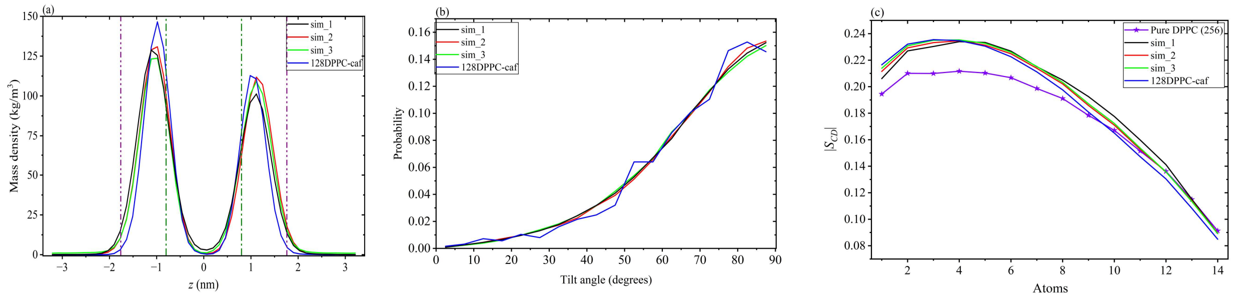

Figure S6. (a) Mass density profiles of caffeine molecules. The purple and olive-green dot-dashed vertical lines show the peak positions in the mass density profile of the headgroup and acyl chains of the DPPC molecules. (b) Probability distribution of tilt angle in the caffeine plane. (c) The Deuterium order parameter (sn-1). Also shown is the corresponding behavior of the pure DPPC + water system. Data presented in panels (a)-(c) correspond to the equilibrated part of the MD trajectory (last 100 ns).

The preferred orientation of caffeine molecules is consistent among all systems studied here and corresponds to a parallel conformation to the acyl chains as shown in panel (b) of Figure S6. The small irregularity observed in the 128 DPPC–caffeine system is likely due to the smaller sample size arising from the reduced number of caffeine molecules. As seen in panel (c) of Figure S6 the order parameter for the equilibrated part of the simulation (last 100 ns) shows similar behavior as the refence system. The order parameter increases near the headgroup atoms, barely affecting the tail region of the acyl chain.

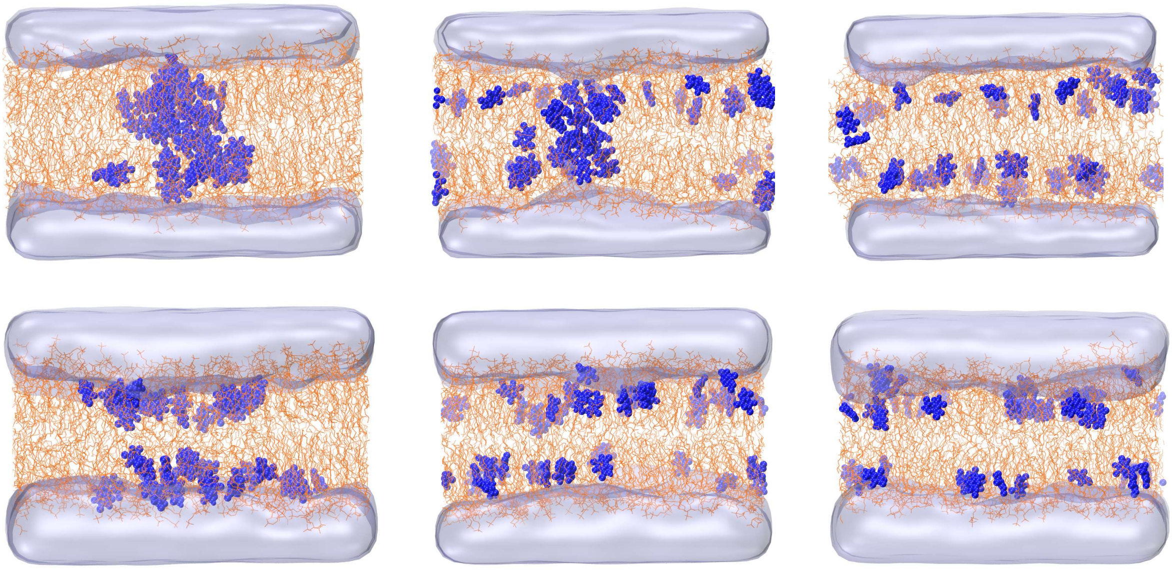

Figure S7. System snapshots at various time instances from two independent simulations, labeled as "sim_4" (top panel) and "sim_5" (bottom panel) where caffeine molecules are placed at the hydrophobic phase. From left to right: t = 1, 500 and 1000 ns ("sim_4") and t = 1, 250 and 500 ns ("sim_5").

Figure S7 shows two independent simulations where caffeine molecules are placed in the hydrophobic phase as a cluster (sim_4) and in a rather dispersed manner (sim_5) initially. The caffeine molecules reach the headgroup-tail interface on equilibration, showing similar behavior as the first scheme, where the caffeine molecules are placed initially in the water.

Two videos showcasing the caffeine permeation in the DPPC membrane corresponding to the two different initial caffeine placements are available in the following link:

Video 1: Caffeine placed initially in the water volume; Video 2: Caffeine placed initially in the hydrophobic core. The videos are composed of frames up to *t* = 800 ns. For visual clarity of the caffeine molecules, the DPPC and water molecules are represented as "points" with orange and cyan colors, respectively. The atoms in the caffeine molecules are shown as blue spheres. The frames of the video have been created through the VMD software (version 1.9.3).

References:

[1] N. Michaud-Agrawal, E. J. Denning, T. B. Woolf, and O. Beckstein, "MDAnalysis: a toolkit for the analysis of molecular dynamics simulations," *J. Comput. Chem.*, vol. 32, no. 10, pp. 2319–2327, Jul. 2011, doi: 10.1002/JCC.21787.

[2] R. J. Gowers *et al.*, "MDAnalysis: A Python Package for the Rapid Analysis of Molecular Dynamics Simulations," *In Proceedings of the 15th Python in Science Conference, Austin, TX, USA, 11–17 July 2016*, pp. 98–105, Sep. 2019, doi: 10.25080/MAJORA-629E541A-00E.
